# Chemical Aging of Semivolatile Secondary Organic Aerosol Sesquiterpene Products

**DOI:** 10.1021/acsestair.5c00011

**Published:** 2025-06-27

**Authors:** Christina N. Vasilakopoulou, Agata Blaziak, Damianos Pavlidis, Angeliki Matrali, Kalliopi Florou, Petro Uruci, Spyros N. Pandis

**Affiliations:** 1 54570 Institute of Chemical Engineering Sciences, FORTH/ICE-HT, Patras GR 26504, Greece; 2 Department of Chemical Engineering, 37795 University of Patras, Patras GR 26504, Greece; 3 Institute of Physical Chemistry, 119463 Polish Academy of Sciences, 01-224 Warsaw, Poland

**Keywords:** biogenic VOCs, β-caryophyllene, δ-cadinene, later-generation SOA, atmospheric simulation chamber, physicochemical mechanism, functionalization probability

## Abstract

The oxidation of biogenic volatile organic compounds (bVOCs) is a significant source of secondary organic aerosols (SOA). This study investigates later-generation SOA formation from the reactions of three first-generation sesquiterpene ozonolysis products (β-nocaryophyllinic acid, 2-(2-carboxyethyl)-3,3-dimethylcyclobutane-carboxylic acid, and 2-(2-methyl-6-oxoheptan-3-yl)-3,6-dioxoheptanal) with the OH radical. These compounds were synthesized specifically for this study. Our results demonstrate that sesquiterpene-derived SOA can become progressively more oxidized as it reacts with OH, with an average oxygen-to-carbon (O:C) ratio of approximately 0.6, in contrast with previous studies, suggesting considerably lower ratios. As the first-generation products continue to react, the corresponding SOA aerosol mass spectrometer (AMS) mass spectrum changes by 20–25° compared to that of the fresh SOA. This suggests that quantifying the sesquiterpene SOA under ambient conditions based on its ozonolysis products may be problematic. To account for these observations, we propose and test a physicochemical model describing the processes that convert first-generation sesquiterpene SOA into highly oxidized compounds. Our analysis suggests that approximately two-thirds of carbon in the ozonolysis SOA follows functionalization pathways. The parametrizations developed in this work can be utilized in future modeling efforts to reassess the contribution of sesquiterpenes to SOA formation.

## Graphical abstract

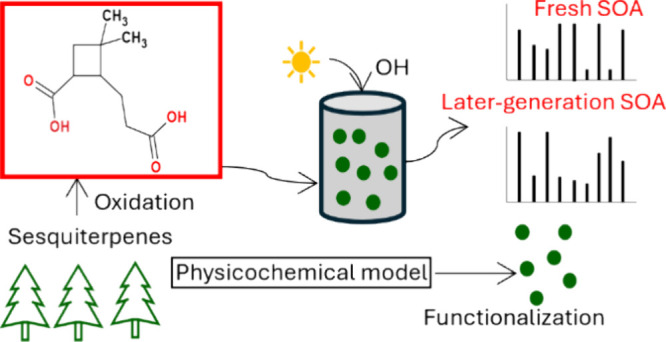

## 1 Introduction

Secondary organic aerosol (SOA) is produced in the atmosphere when oxidation products of organic vapors condense onto preexisting particles. Global SOA production is estimated^1^ at 22–100 Tg y^–1^. Advanced measurements around Europe have indicated that oxidized organic aerosol (OOA) accounts for approximately 70% (range 44–100%) of the total OA.^2^ Biogenic volatile organic compounds (bVOCs), primarily emitted by vegetation, dominate global emissions and react rapidly in the atmosphere, playing a central role in SOA formation.^3,4^ Emissions and reactivity increase with temperature, making bVOC oxidation a major pathway for SOA production, particularly in warmer, forested regions.^5^

The key bVOCs are isoprene (C_5_H_8_), monoterpenes (C_1__0_H_1__6_), and sesquiterpenes (C_1__5_H_2__4_). While SOA formation from isoprene and monoterpenes has been extensively studied,^6–9^ sesquiterpene SOA remains less understood. Sesquiterpenes are emitted by plants as part of defense mechanisms, are highly reactive, and present measurement challenges due to their short atmospheric lifetimes.^4^ Despite their lower emissions, sesquiterpenes yield significantly more SOA than do other bVOCs. Tasoglou and Pandis^10^ reported SOA yields for β-caryophyllene oxidation ranging from 20 to 38% at 10 μg m^–^^3^. Chen et al.^11^ found even higher yields, ranging from 15 to 70% for 2–77 μg m^–^^3^ of OA. Other studies have reported SOA yields of approximately 50% for β-caryophyllene and α-humulene oxidation.^12,13^ A recent study^14^ on α-humulene ozonolysis also reported high SOA yields, ranging from 30 to 70% for 10–100 μg m^–^^3^ of OA.

The above laboratory studies have consistently shown that the resulting first-generation ozonolysis SOA has relatively low oxygen-to-carbon (O:C) ratios (e.g., 0.25–0.53). Tasoglou and Pandis^10^ reported an O:C ratio of 0.25–0.44 for β-caryophyllene SOA using the Aiken et al.^15^ method, and 0.44–0.53 using the more accurate Canagaratna et al.^16^ method. Chen et al.^11^ reported β-caryophyllene SOA O:C ranging from 0.33 to 0.54. Ye et al.^17^ performed experiments of mixed precursors using isotopically labeled VOCs, finding O:C of 0.29–0.31 for β-caryophyllene SOA. Sippial et al.^14^ reported an O:C of 0.25 for α-humulene ozonolysis SOA. However, field studies report higher O:C ratios for biogenic SOA including that derived from sesquiterpenes. For example, studies in the southeastern US and forested sites in Canada, Japan, and Greece report O:C values up to 0.8, attributed to ongoing oxidation processes.^18–23^ Such discrepancies highlight the limitations of laboratory experiments, which often simulate only the initial hours of oxidation, while atmospheric aerosols may persist and evolve over days.^3,24,25^

We hypothesize that the rapid ozonolysis of sesquiterpenes in the atmosphere is followed by swift reactions of their first-generation oxidation products with OH radicals. These subsequent reactions produce more highly oxidized second- and later-generation products that dominate sesquiterpene SOA in the atmosphere, even in forested areas.

In the present study, we synthesized three sesquiterpene oxidation products (Figure 1) and quantified their physicochemical properties and potential for SOA formation. Two β-caryophyllene oxidation products (β-nocaryophyllinic acid (C_13_H_20_O_5_) and 2-(2-carboxyethyl)-3,3-dimethylcyclobutanecarboxylic acid (β-200, C_10_H_16_O_4_)) and one δ-cadinene oxidation product (2-(2-methyl-6-oxoheptan-3-yl)-3,6-dioxoheptanal (δ-284, C_15_H_24_O_4_)) were chosen.^26^ These amphiphilic and surfactant-like compounds have been identified in atmospheric studies.^27–29^ These products were synthesized for the purposes of this study, and this is the first time that they have been used in atmospheric simulation chamber experiments. The investigation of their oxidation allows quantification of the role of later-generation reactions in the sesquiterpene SOA system and its potential for later-generation SOA formation. The results of this study also provide insights into the complexity of the continuously evolving atmospheric SOA and the limitations of the current mental model focusing on the first generation of reactions.

**Figure 1:**
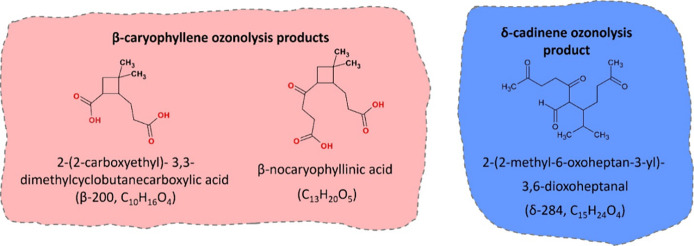
Sesquiterpene ozonolysis products studied.

## 2 Methods

### 2.1 Experimental Procedures

Laboratory atmospheric simulation chamber experiments were conducted in order to study the SOA formation from the reactions of three sesquiterpene first-generation (ozonolysis) products, (β nocaryophyllinic acid, 2-(2-carboxyethyl)-3,3- dimethylcyclobutane carboxylic acid, and 2-(2-methyl-6-oxoheptan-3-yl)-3,6-dioxoheptanal) (Figure 1) with the OH radical. These compounds are known bicyclic sesquiterpene ozonolysis products and could be synthesized at high purity with considerable effort. Their synthesis was conducted in the Institute of Physical Chemistry in Warsaw (Poland). These complex organic molecules could serve as initial examples of the chemical aging that the many similar sesquiterpene ozonolysis products undergo in the atmosphere.

The experimental conditions are listed in Table 1. The experiments were conducted in the FORTH atmospheric simulation chamber (FORTH-ASC). The experimental setup is shown in Figure 2. The 10 m^3^ Teflon chamber is located inside a 30 m^3^ temperature-controlled room surrounded by UV lights.

**Table 1:** Experimental Conditions for Photooxidation Experiments of the Products

exp.	seeds (μg m^–3^)^a^	initial OA (μg m^–3^)	ΔOA (μg m^–3^)^b^	OH (molec. cm^–3^)^c^	O:C (final)^d^	*a* ^e^
**C**_**10**_ **H**_**16**_ **O**_**4**_						
1	43	56				
2	36	11	4.3	3.2 × 10^7^	0.71	0.65
3	20	6.5	5.2	5.2 × 10^7^	–	0.6
4	52	4.5	7.2	3.3 × 10^7^	0.63	0.5
5	55	22.5	2.8	3 × 10^7^	0.60	0.7
**C**_**13**_ **H**_**20**_ **O**_**4**_						
6	24	68				
7	77	297	17.5	3 × 10^7^	0.37	0.9
8	27	11	12.2	4 × 10^7^	0.53	0.7
9	70	21	2.9	1.9 × 10^7^	0.59	0.7
**C**_**15**_ **H**_**24**_ **O**_**4**_						
10	52	24	13.1	4.3 × 10^7^	0.5	0.75
11		107				
12		39	17.2	3.4 × 10^7^	0.36	0.95
13		203	19.7	3 × 10^7^	0.38	0.85

a Ammonium sulfate was injected for the wall loss characterization. In experiments 1–10, the ammonium sulfate was injected prior to the compound injection, while in experiments 11–13, it was injected at the end of the experiment (after cleaning the chamber).

b Increase in OA concentration.

c Average OH during the first hour of oxidation. Exps. 1, 6, and 11 are characterization experiments without oxidation.

d Atomic oxygen-to-carbon ratio after the chemical aging with OH.

e Fraction of the aging proceeding through the functionalization pathway.

**Figure 2:**
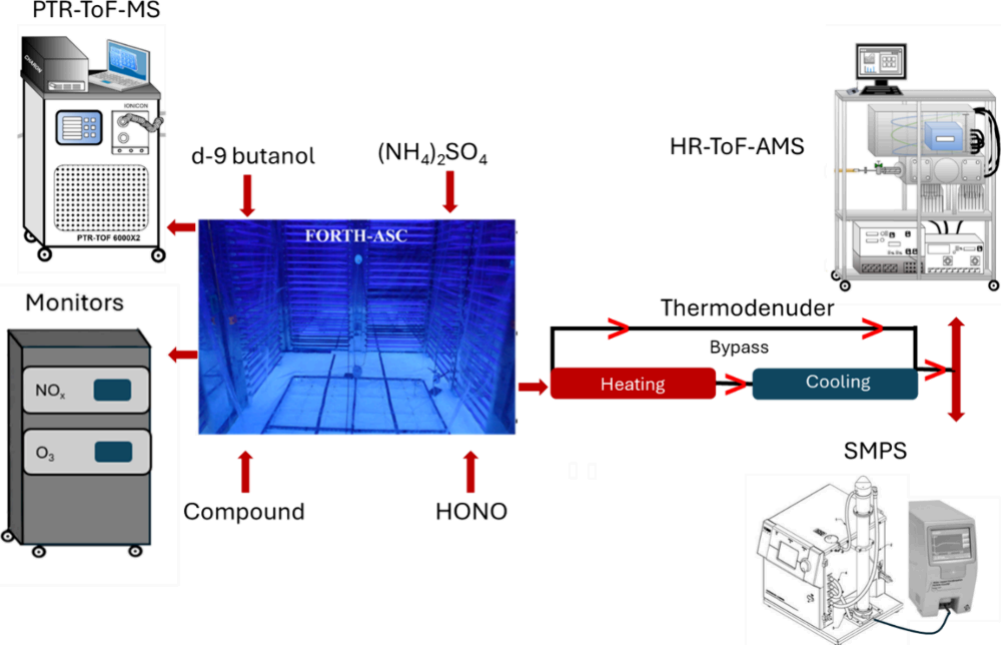
Schematic of the experimental setup used in this study. The instruments for the measurement of the gas phase pollutants (PTR-ToF-MS, O_3_, and NO_*x*_ monitors) together with the instruments for the particle phase (AMS, SMPS) and the system for the SOA volatility quantification (thermodenuder) are shown.

The chamber was cleaned for 30–35 h before each experiment. The cleaning procedure includes flushing with purified air for 15 h, then injection of nitrous acid (with the UV lights turned on to produce OH) for 3 h, and a second flushing stage with clean air for 15 h. Blank experiments were performed every 10 days to ensure clean conditions of the chamber. A blank experiment includes all of the steps of the full experiment without the injection of an organic compound.

Before the start of each experiment, the chamber was filled with clean purified air. Then, ammonium sulfate seeds were injected into the chamber. The injection was performed by using an atomizer (TSI 3076) and a silica gel dryer. The chamber stayed undisturbed for 1.5–2 h for the characterization of the particle losses to the walls. Then, the target organic compound was injected. Some experiments (11–13) used only the target organic compound and no ammonium sulfate particles to avoid the corresponding impurities. In these cases, the wall losses were measured at the end of the experiment after the chamber was cleaned from the organics. The parent compounds in our experiments exist in both the gas and particulate phases. So, there is a significant surface area in the beginning of the experiments for the condensation of any new material. The relative consistency of the experiments with and without seeds is also indirect evidence that the effect of the seeds was negligible.

The organic compound injection was performed using a flash vaporization system due to the low water solubility of these compounds. Due to the physical properties of the precursors (they are highly viscous liquids at room temperature) and the very small volumes used in the vaporizer, the exact amount of compound injected could not be directly measured. Different amounts of the parent compounds were injected in different experiments, resulting in the different initial OA concentrations shown in Table 1. The organic particles were allowed to equilibrate in the chamber, and therefore initially, the parent compound was present in both the gas and particulate phases. D-9 butanol was also added to the chamber, serving as a tracer for OH radicals.^30^

Both characterization and chemical aging experiments took place. In the characterization experiments, the system stayed undisturbed for several hours, during which time the volatility of the OA was measured using a thermodenuder (TD). The time step used between TD measurements and the bypass (line) was 5 min. The TD temperatures ranged from 35 to 80 °C, with 15 min of measurements at each temperature. The mass fraction remaining (MFR) was calculated based on the concentration of the compound corrected for wall losses in the TD, AMS collection efficiency, and thermodenuding artifacts. Positive matrix factorization (PMF) was performed in order to estimate the thermodenuding artifacts present at high temperatures in the OA and to subtract them from the compound concentration remaining after passing through the TD (SI Section S1, Figure S1). In order to estimate the OA volatility, the Riipinen et al.^31^ approach was used. The saturation concentration (C*) and the effective vaporization enthalpy (Δ*H*) were estimated based on the MFR results.

In the chemical aging experiments, HONO was injected into the chamber. In various experiments, the formation process took longer and additional HONO injections were required (e.g., Exp. 4). After the HONO injection, the UV lights were turned on to initiate the production of OH radicals. Most of the experiments were performed at low relative humidity (RH 10–20%). There was one experiment performed in high RH. In this case, we did not observe substantial differences in either the concentration or the oxidation of the SOA compared with the low-RH experiments. While this suggests that humidity did not have a major impact under our specific experimental conditions, we acknowledge that more targeted investigations would be needed to fully assess the role of RH in SOA formation.

The particle phase was continuously characterized using an aerosol mass spectrometer (AMS; Aerodyne Inc.) and a scanning mobility particle sizer (SMPS; TSI). For the AMS data analysis, SQUIRREL 1.65A and PIKA 1.25A were used. A proton transfer reaction mass spectrometer (PTR-MS) (PTR-TOF 6000; Ionicon) and standard gas monitors were used for measurements of the various compounds in the gas phase. In some experiments, unit mass resolution PTR-MS (Ionicon) was used. The size-dependent collection efficiency (CE) was estimated based on the Pavlidis et al.^32^ approach. Briefly, the algorithm matches the volume distribution of the SMPS and the mass distribution by the AMS using a size-dependent collection efficiency and an organic aerosol density. A size-dependent wall loss rate constant, corrected for coagulation, was calculated following the approach of Nah et al.^33^ by utilizing the evolution of the particle size distribution measured by the SMPS from the seeds-only period of the experiment. The results of each experiment were corrected with the corresponding wall loss rate constant measured for that experiment.

Two different types of positive matrix factorization (PMF) analyses were performed in this study. First, PMF analysis was performed in chemical aging experiments to quantify the concentration of the parent compound (sesquiterpene oxidation product) and the later generation SOA. In these experiments, there was no TD operating. PMF was performed separately for each experiment. High-resolution AMS measurements were used for PMF analysis. Additional information about the PMF is included in the Supporting Information. Second, the TD results of the characterization experiment were analyzed by PMF to quantify any artifacts introduced during thermodenuding. In the raw MFR results (Figure S1), there is a right tale at high temperatures, and the mass never goes to zero as it should for a single component aerosol. PMF showed that this odd behavior is due to thermodenuding artifacts that appear at high temperatures. Using PMF, we were able to characterize these artifacts and correct the MFR results.

As a metric of similarity between AMS spectra, we use the theta angle, which is the angle between two spectra that are treated as vectors.^34^ Other names like “spectral contact angle” are used for the same angle in other applications of mass spectrometry (e.g., peptide sequencing). We classify as “similar” spectra with angles below 15° and “different” spectra with angles above 15°.^35^

### 2.2 Aging Mechanism

A physicochemical mechanism was developed in order to explain the experimental results (Figure 3). After their injection into the chamber, the semivolatile sesquiterpene product partitions between the particle and the gas phase, and we assume that equilibrium is rapidly established. This equilibrium partitioning is described by

$$C_{g}(t)=\frac{C_{p}(t)}{C_{\mathrm{OA}}(t)}C^{0}$$ 1

where *C*_g_(*t*) is the concentration of the parent compound (e.g., C_10_H_16_O_4_) in the gas phase, *C* ^0^ is its saturation concentration, *C*_p_(*t*) is the concentration of the same compound in the particle phase, and *C*_OA_(*t*) is the measured total organic aerosol concentration.

**Figure 3:**
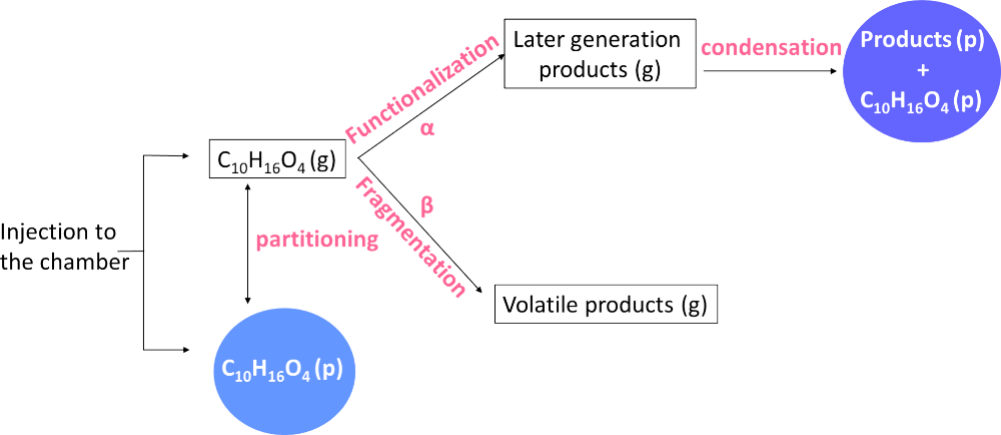
Schematic of the proposed mechanism for later-generation SOA formation during the reaction of C_10_H_16_O_4_ with OH. Similar mechanisms are used for the other two compounds.

The compound is assumed to react with OH radicals in the gas phase to produce a later generation of SOA products. The corresponding mass balance is

$$\frac{dC_{g}}{dt}+\frac{dC_{p}}{dt}=-k[\mathrm{OH}]C_{g}$$ 2

where *k* is the rate constant for the gas phase reaction between the parent compound and OH, and [OH] is the concentration of OH radicals.

The products can condense in the particle phase and produce later generation SOA resulting in the following equation for the SOA concentration:

$$\frac{dC_{\mathrm{SOA}}}{dt}=ak[\mathrm{OH}]C_{g}$$ 3

where *C*_SOA_(*t*) is the concentration of the produced later generation SOA and *a* is the yield for the produced nonvolatile later generation SOA.

Finally, the total OA is given by the following mass balance:

$$C_{\mathrm{OA}}(t)=C_{\mathrm{SOA}}(t)+C_{p}(t)$$ 4

Since the gas phase concentration of the injected compound is reduced, part of the particle phase evaporates to maintain equilibrium, and the SOA production in the gas phase continues. In this work, we define fragmentation as the set of reactions that cause significant reductions in the size of the molecule, increasing significantly its volatility and leading to its transfer to the gas phase. Reactions that lead to the loss of a small fragment from a large molecule (e.g., C15) would not be effectively counted as fragmentation by using this definition.

The assumptions used in this mechanism are that the parent compound does not interact with the preexisting inorganic aerosol, there is continuous rapid equilibration between the gas and the particle phases of the parent compound, the original and final organic compounds have similar molecular weights,^36^ the products of the oxidation have low enough saturation concentration so they are present only in the particle phase, and finally the heterogeneous reactions do not play a significant role.

The systems of eqs 1–4 can be converted to the following system of differential equations (SI, Section S2):

$$\frac{dC_{g}}{dt}=-\frac{k[\mathrm{OH}]+\frac{1}{C^{o}}\frac{dC_{\mathrm{OA}}}{dt}}{\frac{C_{\mathrm{OA}}}{C^{o}}+1}C_{g}$$ 5

$$\frac{dC_{\mathrm{SOA}}}{dt}=\alpha k[\mathrm{OH}]C_{g}$$ 6

$$\frac{dC_{p}}{dt}=\frac{dC_{\mathrm{OA}}}{dt}-\alpha k[\mathrm{OH}]C_{g}$$ 7

using the following initial conditions:

$$C_{g}\left(0\right)=C^{0}$$ 8

$$C_{\mathrm{SOA}}\left(0\right)=0$$ 9

$$C_{p}\left(0\right)=C_{\mathrm{OA}}\left(0\right)$$ 10

*C*_OA_(*t*) and d*C*_OA_(*t*)/d*t* can be obtained directly from the measurements during each experiment. A value of 3 × 10^–7^ cm^3^ molecule^–1^ h^–1^ has been estimated^37^ for the rate constant *k* and is used in this case. The OH concentration was calculated based on the d9-butanol decay. The *C* ^0^ is measured using the thermodenuder.

The system of eqs 5–7 was solved numerically using varied values of the parameter alpha (α) between 0 and 1 using the explicit Runge–Kutta method of order 5. The best fit was chosen, based on the ability of the model to estimate the concentration of the compound and that of the later-generation SOA. The errors of the model contain the error in the rate constant and in the derivative of the organic aerosol. However, the ability of the model to reproduce the rate of increase in the later-generation SOA given by the PMF is reassuring. The assumed reaction rate constant mainly determines the slope of the concentration curve and not the final concentrations after 1 h or so. Therefore, our main results are not sensitive to the estimated reaction constant value. The model was used to simulate only the first 3 h, without the additional injection of HONO, because then a new set of equations, parameters, and conditions would be needed to simulate the chamber measurements. The measurements were corrected for the impurities from the ammonium sulfate seeds; when running the model, the correction was minor though. Even high-purity ammonium sulfate can contain traces of organic contaminants.^38^

## 3 Results and Discussion

### 3.1 Volatility Measurements

In the first stage of this study, characterization experiments were performed in order to quantify the volatility of each compound as a function of the temperature. In these experiments, the parent compound was injected into the chamber in the dark, without the addition of an oxidant. A thermodenuder (TD) was used in these experiments.^39^

The MFR of the C_10_H_16_O_4_ in Exp. 1 is shown in Figure 4. According to the model, there is some evaporation at room temperature in the cooling section of the TD, which is filled with activated carbon and removes organic vapors from the sample. As a result, the MFR is not equal to unity at room temperature. The estimated *C* ^0^ at 298 K using these data was 10 μg m^–3^, and the effective Δ*H* of evaporation was 50 kJ mol^–1^. The compound *C* ^0^ at 298 K was also estimated theoretically using existing vapor pressure estimation methods^40–43^ and varied between 3 and 20 μg m^–3^. These relationships are quite uncertain for these large, highly oxygenated complex molecules. Thermodenuder experiments were performed for the other two compounds as well, giving a saturation concentration of 6 μg m^–3^ for C_13_H_20_O_5_ and 17.6 μg m^–3^ for C_15_H_24_O_4_ at 298 K. The TD was used in selected experiments only to quantify the volatility of the precursor, which is needed as an input to our model. The study of the volatility distribution of the products needs to be a part of future work. The relatively long residence times used in our TD allowed us to use temperatures less than 60 °C for the practically complete evaporation of the compounds under investigation. With this modest heating, we assume that thermal degradation had a minimal effect, if any, on our measurements.

**Figure 4:**
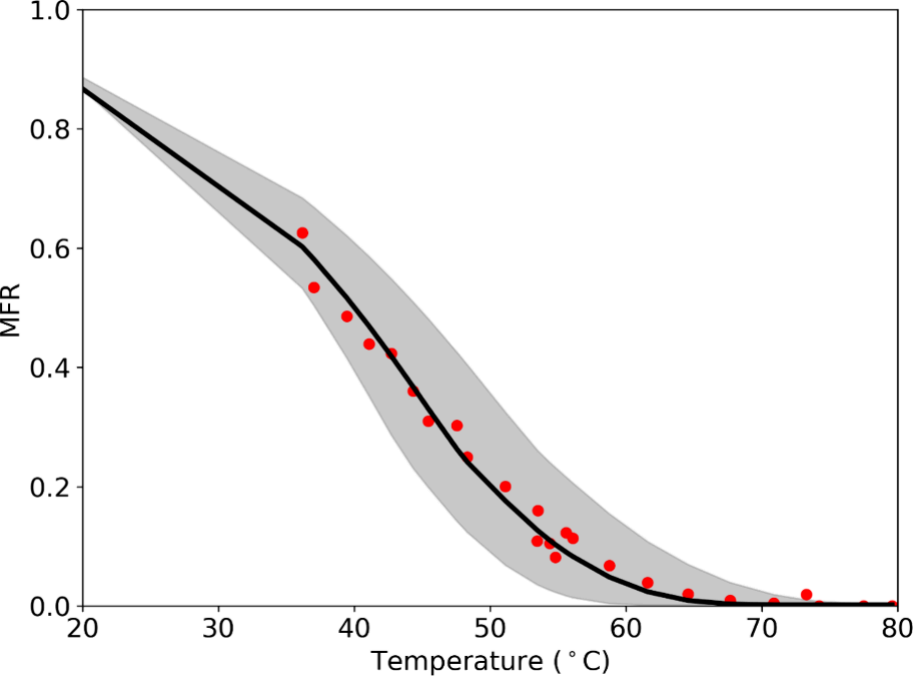
Mass fraction remaining as a function of thermodenuder temperature for C_10_H_16_O_4_ (Exp. 1). The symbols are the measurements, and the black line represents the model predictions. The shaded region corresponds to the model prediction uncertainty.

### 3.2 AMS Spectra of the Compounds

The organic AMS mass spectrum of C_10_H_16_O_4_ was characterized by *m*/*z*’s 41 (C_3_H_5_^+^), 44 (CO_2_^+^), 56 (C_4_H_8_^+^), 67 (C_5_H_7_^+^), 77 (C_6_H_5_^+^), 79 (C_6_H_7_^+^), and 91 (C_7_H_7_^+^) (Figure 5). These ions had a high relative intensity. Typically, *m*/*z*’s 28 and 44 are observed in oxidized spectra, while *m*/*z*’s 41, 55, and 67 are observed in hydrocarbon-like OA and *m*/*z* 91 has been observed in biomass burning OA. The strong peak in *m*/*z* 56 (mainly C_4_H_8_^+^) is a possible tracer for this compound. In the high *m*/*z*’s (above 100), some interesting peaks were also observed at *m*/*z*’s 127 (C_6_H_7_O_3_^+^), 136 (C_8_H_8_O_2_^+^ and C_9_H_12_O^+^), and 164 (C_8_H_4_O_4_^+^) (Figures S6–S8). The fractional spectra for all experiments with the same precursor were very similar to each other (Figure S16).

**Figure 5:**
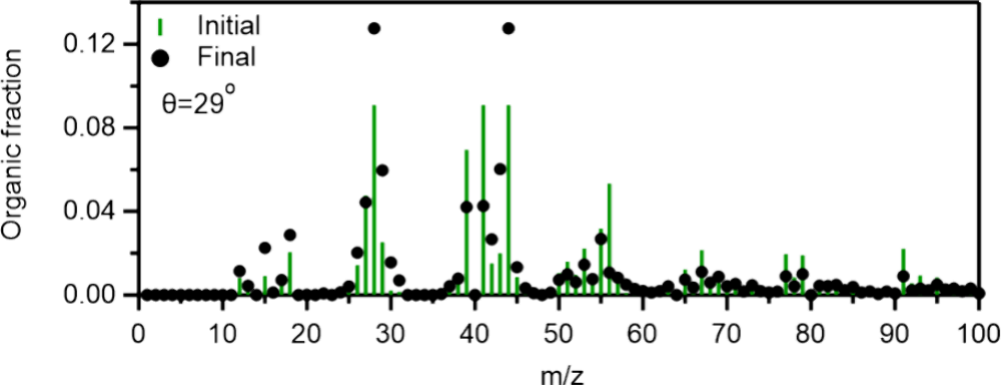
Comparison of the initial (−0.5 to 0 h) and final (4 to 4.5 h) organic AMS spectra during the C_10_H_16_O_4_ photooxidation experiment 2. The AMS data were processed at high mass resolution and then were summed to unit resolution to produce the figures.

The β-norcaryophyllinic acid OA spectrum (C_13_H_20_O_5_) was characterized by the *m*/*z*’s 53 (C_4_H_5_^+^), 56 (C_4_H_8_^+^), 67 (C_5_H_7_^+^), 77 (C_6_H_5_^+^), 79 (C_6_H_7_^+^), 91 (C_7_H_7_^+^), 95 (C_7_H_11_^+^ and C_6_H_7_O^+^), 115 (C_9_H_7_^+^), 137 (C_8_H_9_O_2_^+^ and C_9_H_13_O^+^), and 165 (C_13_H_9_^+^) (Figure S5). The *m*/*z*’s 91, 115, 137, and 165 have been reported in biomass burning spectra in the literature, with the 91, 115, and 137 being identified in spectra produced by the combustion of lignin, while 165 has been found in dung burning.^44^ The *m*/*z* 91 is also a marker of monoterpene SOA^45^ and isoprene SOA.^46^ The *m*/*z*’s 115 and 165 have also been related to polycyclic aromatic hydrocarbons (PAHs).^47^ The O:C of the β-nocaryophyllinic acid spectrum was 0.34 ± 0.06 for the experiments performed.

The C_15_H_24_O_4_ spectrum was dominated by *m*/*z* 43 (C_2_H_3_O^+^ and C_3_H_7_^+^), while smaller peaks were also observed at *m*/*z*’s 41 (C_3_H_5_^+^), 69 (C_5_H_9_^+^ and C_4_H_5_O^+^), 77 (C_6_H_5_^+^ and C_3_H_9_O_2_^+^), 79 (C_6_H_7_^+^), 81 (C_6_H_9_^+^ and C_5_H_5_O^+^), 91 (C_7_H_7_^+^), 173 (C_13_H_17_^+^ and C_12_H_13_O^+^), and 191 (C_15_H_11_^+^). The major *m*/z 43 peak has also been observed in sesquiterpene oxidation experiments.^10^ The AMS O:C was 0.28.

### 3.3 Chemical Aging of C_10_H_16_O_4_

The results of experiment 2, in which the photooxidation of C_10_H_16_O_4_ was studied, are shown in Figure 6. The compound was injected into the dark chamber and was distributed in both the particle and gas phases. While the initial OA concentration was 10 μg m^–3^, the initial gas phase concentration was 9.5 μg m^–3^ assuming that it was equal to its saturation concentration at that temperature.

**Figure 6:**
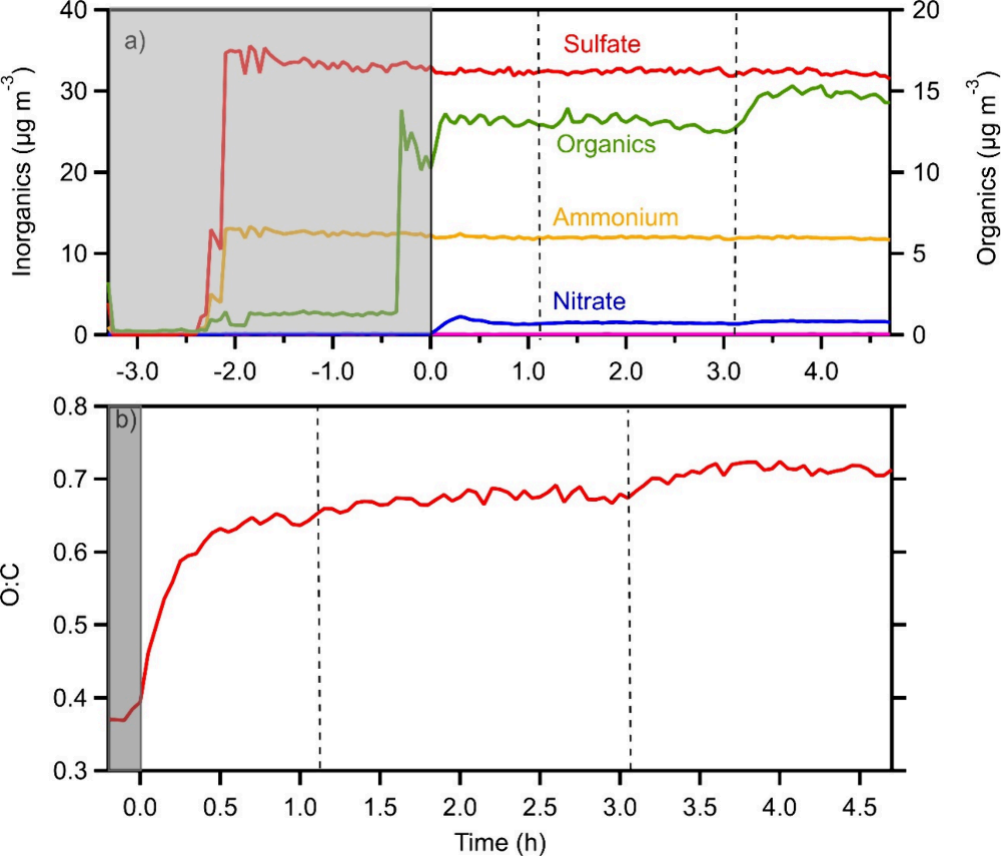
Results of C_10_H_16_O_4_ aging experiment 2: (a) concentrations of the aerosol components. Different axes are used for the inorganics (left) and the organics (right); (b) time series of the O:C. The gray area represents the dark period of the experiment. The dashed lines represent the injections of HONO. The OA increase in approximately 3 h is due to the addition of HONO. The *x*-axis is different for the two graphs, so that only the O:C of the studied chemical system is shown.

After the UV lights were turned on (time zero) and OH was produced by HONO photolysis, the OA concentration in the chamber increased to 15 μg m^–3^ (Figure 6). The OH concentration was 3.2 × 10^7^ molecule cm^–3^. Three HONO injections were performed in this experiment, at 0 h, at approximately 1 h, and at 3 h. The change of the OA, as we will see later, is the result of both the production of later-generation SOA, but also the consumption of C_10_H_16_O_4_. After approximately 1 h, practically all the later-generation SOA was formed in all experiments. A small increase in nitrate (1 μg m^–3^) was also observed (80% organonitrates), after turning the UV lights on, because of the high NO_*x*_ levels (SI Section S3). The NO_*x*_ concentration in experiment 2 at the end of the first photooxidation period (after 1 h of oxidation) was 185 ppb. The NO_*x*_ concentration changed during the experiment due to additional HONO injections and ongoing photochemical processes. Specifically, NO_*x*_ increased from 185 to approximately 230 ppb during the second hour and reached 270 ppb by the end of the third hour. The NO_*x*_ concentration of all of the experiments is shown in Table S1.

During the photooxidation, the O:C ratio of the OA increased. In the dark when only C_10_H_16_O_4_ was present, it was 0.38 (the O:C of the compound is 0.4), and it almost doubled at the end of the experiment, reaching approximately 0.7 (Figure 6). O:C reached almost 0.65 in the first 30 min of the experiments, showing that the chemical aging is relatively fast. In this case, this 30 min exposure to OH corresponds to 3–6 h of summertime ambient conditions (OH = 5 × 10^6^–1 × 10^7^ molecules cm^–3^).

The OA AMS spectrum changed by 29° after aging (Figure 5). The major differences were observed for *m*/*z* values of 43 (C_2_H_3_O^+^) and 44, which increased due to aging. *m*/*z* 44 is a known fragment that increases during OA aging. On the other hand, *m*/*z* values 41, 56, 77, 79, 91, 127, 136, and 164, which were relatively high in the original C_10_H_16_O_4_ spectrum, decreased after the oxidation by OH. The decrease of the *m*/*z* 56 signal (C_4_H_8_^+^) may explain why this fragment has not been observed in previous studies in forested areas. The final SOA spectrum differed by 13° with the biogenic OOA spectrum observed in a Greek forest with high emissions of sesquiterpenes.^23^ The fact that the oxidized spectrum from these compounds is moving toward the field measurements shows that the degree of oxidation performed in these experiments is representative of the oxidation that is happening in the field. That is not always true for most chamber experiments, in which we see large differences with the field data. Additional information about the rest of the experiments is shown in SI, Section S4.

Positive matrix factorization (PMF) analysis of the measured OA AMS spectra was performed. The PMF results were corrected for the AMS collection efficiency and particle wall losses. Solutions using one to five factors were explored. The three-factor solution was selected, as it was the one that explained well the measured OA. The residual of the PMF analysis (difference between the measured and the reconstructed OA) was 3% (Figure S17). The PMF results for experiment 2 of C_10_H_16_O_4_ are shown in Figure 7.

**Figure 7:**
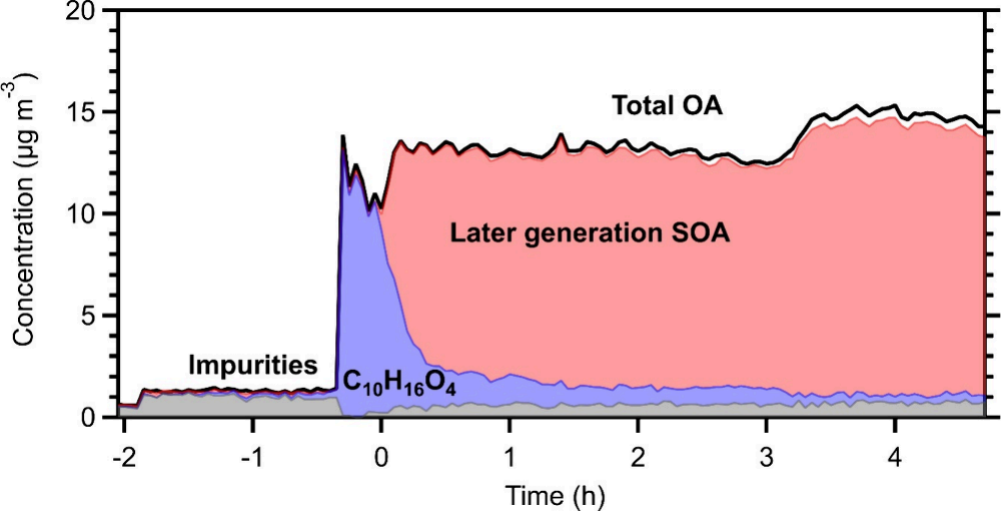
PMF results of the C_10_H_16_O_4_ photooxidation (Exp. 2). The total measured organic aerosol is shown in black, and the three factors are shown in colors (C_10_H_16_O_4_ in blue, later-generation SOA in red, and impurities resulting from the ammonium sulfate seeds in gray).

The first factor increased during the injection of the inorganic seeds and remained relatively constant at 1 μg m^–3^ during the experiment, with the exception of the injection period in which the chamber was not well mixed. This factor represents the organic impurities introduced in the chamber together with the seed particles (SI Section S5). The noisy variation of the impurities at time zero (Figure 7) is mainly due to mixing effects during the introduction of the precursor (and the associated clean air) at a point in the chamber near the sampling point. This incomplete mixing is also observed in the precursor concentration measurements (they decreased for a few minutes, and then they increased). The second factor increased during the injection of the compound into the chamber and decreased after turning on the UV lights. The second factor is the C_10_H_16_O_4_ precursor. The third factor increased while the levels of the precursor started to decrease; thus, it represents the produced later-generation SOA. The AMS spectrum of the SOA factor differed by 32° from that of C_10_H_16_O_4_ (Figure S18). The PMF results indicate that the precursor concentration in the particle phase was greatly reduced (83% reacted) during the first 1 h of the experiment. At the same time, the later-generation SOA started to increase and reached 12 μg m^–3^ after 30 min of oxidation. After approximately 1 h, practically all the later-generation SOA had been formed in all experiments. The O:C of the precursor factor was 0.35, similar to the O:C in the beginning of the experiment (0.38). The later-generation SOA factor had an O:C equal to 0.69, which was also the O:C of the final total organic aerosol. At approximately 3 h, we injected again HONO and the total OA (and the SOA factor) increased. This addition did not result in a new PMF factor different from that of the later-generation SOA. Additional information about the PMF analysis is available in the Supporting Information (SI).

The net increase in the SOA was 2.8–7.2 μg m^–3^ in the different experiments of C_10_H_16_O_4_ oxidation. The later-generation SOA concentration at the end of the experiments was higher and varied between 10 and 20 μg m^–3^. That is because at the end of the experiment, all the initial OA had been transformed to later-generation SOA. The final O:C in the different experiments was 0.65 ± 0.06. This is relatively high compared with values found in the literature for SOA produced during the oxidation of sesquiterpenes. It suggests that in our case, we were able to produce much more oxygenated later-generation products. The time series and spectra of the PMF solution of all experiments are shown in the SI.

### 3.4 Simulations of the Gas and Particle Phase during the C_10_H_16_O_4_ Oxidation

The simulated concentration in the gas and particle phases and the produced later-generation SOA concentration for experiment 2 are shown in Figure 8. The OH concentration for this experiment was 3.2 × 10^7^ molecules cm^–3^. The alpha value that allowed the model to better reproduce the OA measurements was 0.65. The alpha value can be viewed as a potential yield of the later generation of SOA. The rest of the products remain in the gas phase. The results of the model suggest that 90% of the total C_10_H_16_O_4_ (gas and particles) reacted in approximately 1 h. The model results are consistent with the PMF results (Figure 8). This agreement increases our confidence in the results of both approaches. Furthermore, it demonstrates that PMF can be a useful tool in the analysis of experiments in which the initial reactant is a single first-generation product and the subsequent chemistry proceeds rapidly. The predicted concentration of the particle phase *C*_p_ is affected by the noise of the experimental data because its derivative is dominated by the measured d*C*_OA_/d*t*. The small increase in the particle phase concentration (at approximately 1.5 h) is mainly due to small errors in the wall loss correction of the total OA. The reduction in the predicted gas phase concentration was consistent with the measured gas phase concentration of the precursor by PTR-MS. The model uses the measured OA (from the AMS) to calculate the particle phase concentration of the compound and the SOA concentration, so a comparison with the measurement data is not meaningful.

**Figure 8:**
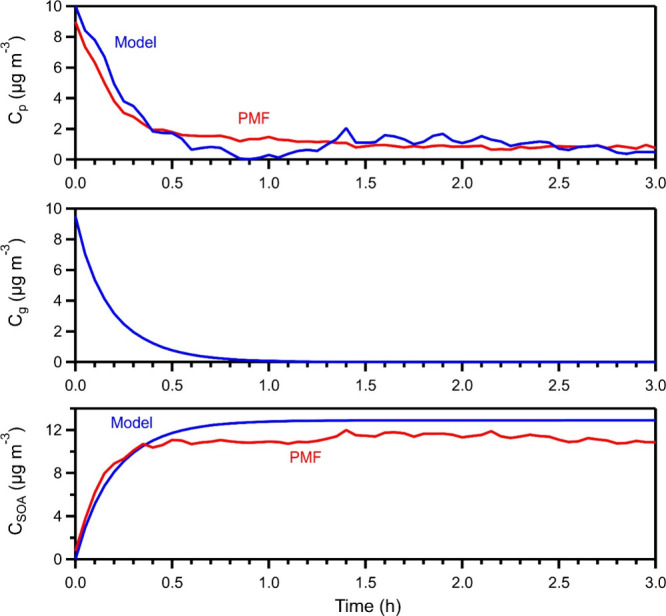
Comparison of the simulated concentrations (in blue) from the physicochemical model describing the aging mechanism (eqs 5–7) and the concentrations obtained from the PMF analysis of the AMS measurements (in red) for the particle phase precursor (*C*_p_) and the produced later-generation SOA (*C*_SOA_), and the gas phase precursor (*C*_g_) (Exp. 2). The alpha value was equal to 0.65.

The PTR-ToF-MS was used in experiment 5, for the measurement of the C_10_H_16_O_4_ gas and particle phase concentration. The results showed that the particle phase half-life time of the studied compound was approximately 20–25 min, which is consistent with the model results (Figure S10). The measured initial C_10_H_16_O_4_ gas phase concentration was approximately 1 ppb (equal to 9 μg m^–3^), which is consistent with the estimated saturation concentration (Figure S10). Due to the high fragmentation of the various large, oxygenated molecules during their protonation inside the PTR-MS, it was practically impossible to use the corresponding PTR-MS measurements to directly estimate the contribution of the functionalization and fragmentation pathways.

The model was utilized for all of the performed C_10_H_16_O_4_ experiments. The alpha value that best matched the OA measurements was 0.61 ± 0.08, which shows that the functionalization pathways dominated. This relatively high value of alpha can explain the high concentration of later-generation SOA that was produced in the experiments performed. If we assume that all the later-generation SOA formed is due to functionalization reactions and the rest (that leads to products that remain in the gas phase) are fragmentation reactions (Figure 3), then the fragmentation probability can be written as β = 1 – α. In our case, β was equal to 0.39, which is much lower than the value of 0.85 calculated by the empirical equation introduced by Chacon-Madrid et al.^48^ The overestimation of the fragmentation using this rough approach has also been previously reported by Karnezi et al.^49^ who simulated the atmospheric organic aerosol during the PEGASOS 2012 campaign.

### 3.5 Chemical Aging of C_13_H_20_O_5_ (β-Nocaryophyllinic Acid)

Approximately 3–18 μg m^–3^ of additional OA was produced during the oxidation of C_13_H_20_O_5_ in the performed experiments (Table 1). The final OA AMS spectrum differed by 17° from the β-nocaryophyllinic acid spectrum. The differences mostly occurred due to the increase of the *m*/*z*’s 28, 43, and 44, and the decrease of the *m*/*z*’*s* 41, 53, 55, 67, 77, 79, 91, 95, 115, 137, and 165 (Figure S12). The O:C of the final OA was 0.56 ± 0.04 for the performed experiments.

PMF analysis was also performed in these aging experiments. A similar behavior with the C_10_H_16_O_4_ results was observed. The precursor concentration in the particle phase was reduced by a factor of 2 during the first hour of oxidation, while in the end of the experiment (3 h), it was practically zero. The OA after 3 h of oxidation was transformed completely to later-generation SOA (Figure S19). The AMS spectra of the three factors can be found in Figure S20. The C_13_H_20_O_5_ SOA spectrum differed by 14° from the C_10_H_16_O_4_ SOA spectrum. The O:C in each experiment is consistent with the relative contributions and O:C values of the PMF-resolved factors (Figures S3 and S14). In nearly all experiments, SOA is the dominant fraction of the organic aerosol by the end of the experiment, resulting in higher overall O:C values. In experiments 7 and 13, a significant fraction of the precursor OA remains at the end, which lowers the overall O:C compared to the others, as the precursor factor has a lower O:C.

Functionalization reaction pathways played a significant role in the chemical aging of C_13_H_20_O_5_, as they were responsible for almost 80% of the conversion. The remaining 20% of the precursor underwent fragmentation. The alpha value that led to the best model fit of the gas and particle phase levels of C_13_H_20_O_5_ and the produced SOA was equal to 0.77 ± 0.08. Estimations of the fragmentation probability based on the O:C of the parent compound based on Chacon-Madrid et al.^48^ suggested a much higher value (0.85 instead of 0.23). So, once more the (O:C)^1/6^ parametrization significantly overestimates fragmentation in this case.

### 3.6 Chemical Aging of C_15_H_24_O_4_

C_15_H_24_O_4_ was the δ-cadinene product used in this work. After its oxidation, approximately 13 μg m^–3^ of OA was produced (exp. 10). The OA spectrum changed by 20° (theta angle), with a clear reduction in *m*/*z* 43. However, this fragment was still significant for the oxidized OA spectrum. The signal at *m*/*z*’s 173 and 191 decreased and the *m*/*z* 44 (CO_2_^+^) increased after oxidation.

The O:C of the final OA was 0.36 (experiment 12), which is relatively low compared to the values observed during the oxidation of the β-caryophyllene products. The average O:C of the oxidized C_15_H_24_O_4_ in the different experiments was 0.41 ± 0.08.

The proposed mechanism for chemical aging was applied to this compound, and the results were evaluated by comparison to PMF analysis. As with the other compounds, functionalization processes dominated over fragmentation, with an alpha value of 0.85 ± 0.1.

### 3.7 SOA Production during the Oxidation of the Sesquiterpene Products

A significant amount of SOA was produced during the OH oxidation of all three sesquiterpene products investigated in this study. The increase in the OA, based on our model, is due to the consumption of the precursor that is originally in the gas phase and in equilibrium with the particulate phase. In all experiments, practically the same amount of gas reacted (equal to the saturation concentration of each compound). If the initial OA concentration is low, then this additional OA will lead to a higher fractional OA increase compared to the experiments in which the initial OA is high. This is the main reason for the observed different percentage changes in OA. The SOA concentration after 2–3 h of oxidation in the chamber was clearly higher compared to the ΔOA. That is due to both the rapid gas phase reaction and the evaporation of the compound in the particle phase. Among the three compounds, the δ-cadinene product (C_15_H_24_O_4_) produced the highest SOA. This is consistent with its higher volatility. Between the two β-caryophyllene products (C_10_H_16_O_4_ and C_13_H_20_O_5_), the latter produced the highest SOA mass. The OH exposure and the reacted concentration of each precursor were similar in the different experiments.

The first-generation ozonolysis products were converted to later-generation products in around 1 h. These results indicate that reactions with OH are a significant process for sesquiterpene SOA formation after ozonolysis takes place. These reactions are not considered in chemical transport models, which may lead to an underestimation of the biogenic SOA.

The O:C of the organic aerosol after the oxidation of the sesquiterpene products by OH was 0.35–0.71. Among the three compounds, the one with the lower molecular weight (C_10_H_16_O_4_) produced SOA with the higher final O:C ratio. That may be due to the need for higher oxidation for the transfer of these products to the particulate phase. A reverse order between the α value and the O:C ratio was observed for the three studied compounds, which suggests that other factors like the size and structure of the molecule may be even more important than the α value. Of course, it is difficult to generalize such a result from just three molecules that are quite different from each other. The final O:C of the C_10_H_16_O_4_ later-generation SOA was similar to that of the biogenic OOA (bOOA) (0.65) observed during the SPRUCE-22 campaign.^23^

The additional oxidation resulted in more aged SOA, which progressively became similar to the biogenic SOA found in the field, as shown in the *f*_44_ vs *f*_43_ triangle plot (Figure 9). The triangle plot proposed by Ng et al.^50^ illustrates the degree of oxidation of organic aerosols (OA). In general, the more oxidized the OA, the further up and to the left it appears in the triangle plot. The additional oxidation moved the chamber OA points inside the triangle area of the field data. However, the chamber SOA did not actually reach the area of ambient bOOA (indicated in the figure with an orange symbol). That is partially because the bOOA consists of compounds that originated from many biogenic VOCs in the atmosphere and not only the three studied here. Additional oxidation may also be possible. This interpretation is not meant to suggest that the oxidation of the selected compounds fully explains the entire ambient sesquiterpene (SQT) profile observed in the field, where multiple sources contribute. Chamber experiments typically exhibit a gap in oxidation compared to field observations, often due to the absence of later-generation oxidation processes. This gap is the motivation for our study, as illustrated in Figure 9, in which some first-generation sesquiterpene SOA components (these are the compounds studied) show lower oxidation than ambient measurements in forests with SQT emissions. By including additional oxidation steps, we approach more realistic oxidation states than observed in the field. Regarding the forest itself, it emits (among others) significant amounts of SQTs.

**Figure 9:**
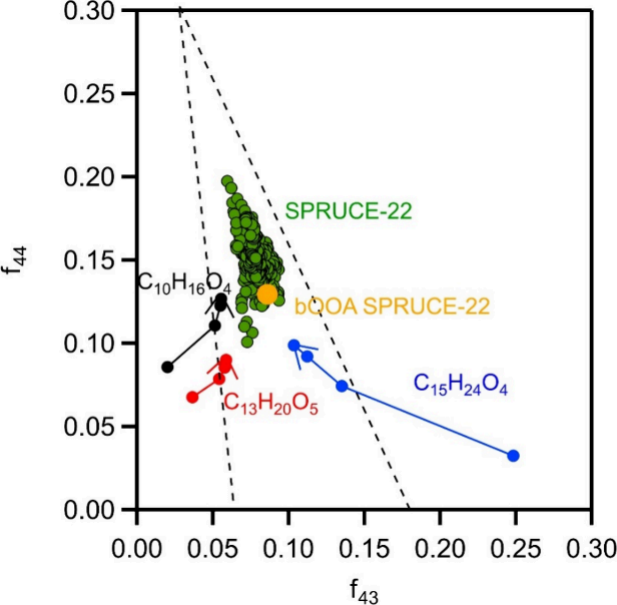
*f*_44_ vs *f*_43_ triangle plot during the oxidation of the three sesquiterpene products. The arrows depict the path of oxidation. The symbols in each path represent the average values at the end of each oxidation period from all experiments of each compound (first symbol for the beginning of the experiment, second symbol for the end of the first oxidation period, third symbol for the second period, and last symbol for the third period). The measurements from SPRUCE-22 and the biogenic SOA factor (bOOA) from the same campaign are also shown.

The produced SOA AMS spectrum was relatively similar for the two β-caryophyllene products (θ = 14°) and differed by 17–18° from the δ-cadinene product. The major differences occurred in *m*/*z* values 28, 43, and 44 (Figure S12). C_10_H_16_O_4_ SOA had higher peaks at *m/z’s* 28 and 44 compared to the rest, while C_15_H_24_O_4_ showed high *m*/*z* 43. The average final SOA organic spectrum of the three compounds differed by 12° from the bOOA spectrum (Figure S13) obtained in a forest during the field campaign SPRUCE-22.^23^

The O:C ratio of the organic aerosol after oxidation of the sesquiterpene products by OH was 0.54 ± 0.12. This is higher than the O:C of the original first-generation compounds (0.35 on average) and of the SOA produced during the ozonolysis of the corresponding sesquiterpene precursors (0.37 on average) found by other studies. The average later-generation SOA spectrum was similar (12°) to that of the biogenic OOA obtained in a forest during the field campaign SPRUCE-22. The compound with the lowest angle with the bOOA from SPRUCE-22 was C_10_H_16_O_4_, which differed by 13°.

The proposed mechanism of the later-generation SOA production includes partial evaporation of the first-generation particles, gas phase oxidation of the resulting vapors by OH, condensation of the produced vapors, and further evaporation of the original particles. This mechanism was able to explain the total OA observations, as it was consistent with the PMF results. The model suggests that the first-generation products reacted in 30–60 min with the OH and later-generation SOA were produced quickly in the system. This is expected to happen in a few hours in an ambient atmosphere.

The functionalization processes seemed to dominate the OH reactions. The average alpha value ranged between 0.5 and 0.95 for these compounds. This shows that the estimation of the fragmentation probability using the current empirical formulas may overestimate the importance of this pathway.

Our study has significant implications for the atmospheric sesquiterpene SOA. The first-generation ozonolysis products react rapidly and are converted to more oxidized compounds. Therefore, these first-generation products are not expected to be found in significant concentrations, even in forested areas. Estimating the SOA from sesquiterpenes from the abundance of their ozonolysis products may lead to a significant underestimation.

## Supplementary Material



## Notes

(Section S1) Mass fraction remaining (MFR); Correction of the MFR data for TD artifacts; (Section S2) calculations with the equations of the proposed model for the SOA production; (Section S3) organonitrate calculation; (Section S4) results of all chemical aging experiments; time series of PM_1_ components, elemental ratios, spectra of each compound, spectra of the SOA from each compound, OH exposure, and NOx concentration per experiment; (Section S5) PMF analysis of chemical aging experiments; time series of the PMF factors of all experiments; time series of the residuals of the PMF per experiment; PMF spectra; and theta angles among all spectra in the paper (PDF)
