# Supplementary material for: Chemical Aging of Semivolatile Secondary Organic Aerosol Sesquiterpene Products
Source: ACS EST Air. 2025 Jun 27;2(7):1180–90. doi: 10.1021/acsestair.5c00011 (PMC12261276; doi:10.1021/acsestair.5c00011)
Supplement: Supplementary file 1 [file ea5c00011_si_001.pdf]

Supplementary Material of:

Chemical aging of semi-volatile secondary organic aerosol sesquiterpene products

Christina N. Vasilakopoulou, Agata Blaziak, Damianos Pavlidis, Angeliki Matrali, Kalliopi Florou, Petro Uruci and Spyros N. Pandis

S1. Mass Fraction Remaining (MFR)

The AMS results after the TD were analysed with Positive Matrix Factorization (PMF) to quantify any artifacts introduced during thermodenuding. In the initial MFR (Figure S1c) there is a right tale at high temperatures, and the mass never goes to zero as it should. PMF showed that this odd behavior is due to thermodenuding artifacts present at high temperatures (Figure S1a, b). Using PMF we were able to characterize these impurities and correct the results. The corrected MFR was used into estimate the volatility of the compounds.

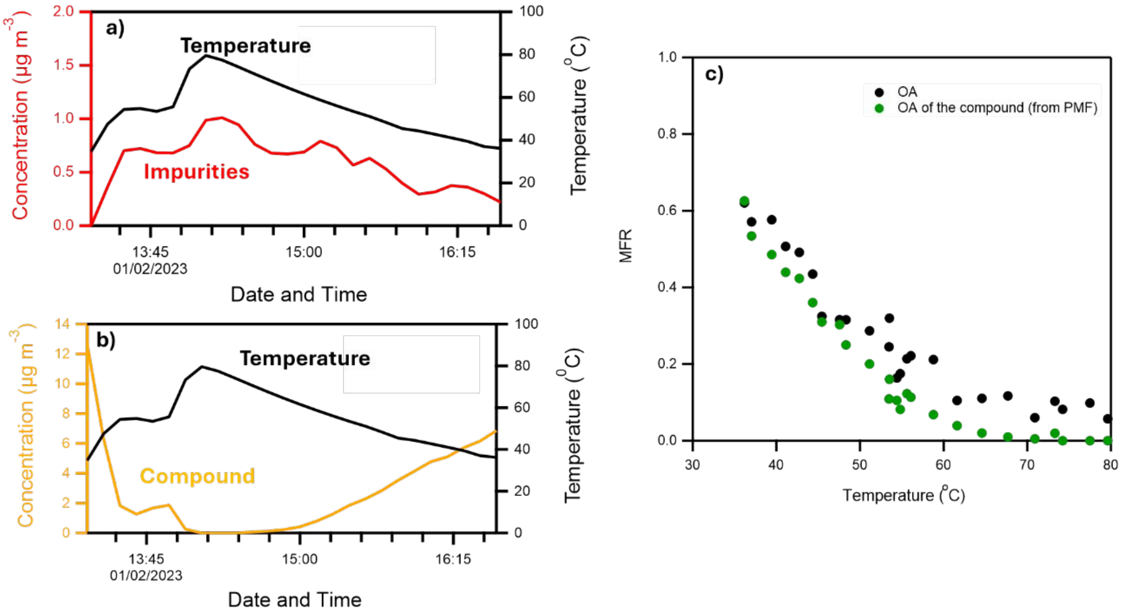

Figure S1: Correction of the MFR data for the TD impurities. a) Impurities timeseries obtained by PMF analysis and TD temperature. b) Compound timeseries obtained by PMF analysis and TD temperature. c) Raw OA (in black) and corrected OA (in green) experimental MFR data.

S2. Equations of the proposed model for the SOA production

Combination of (eq. 1) and (eq. 2):

$$\frac{dc_g}{dt} + \frac{dC_{OA}}{dt}\frac{c_g}{c^0} + \frac{dc_g}{dt}\frac{C_{OA}}{c^0} = -k[OH]c_g \rightarrow$$

$$\frac{dc_g}{dt}\left(1 + \frac{C_{OA}}{c^0}\right) = -k[OH]c_g - \frac{dC_{OA}}{dt}\frac{c_g}{c^0} \quad \rightarrow$$

$$\frac{dc_g}{dt} = -\frac{\left(k[OH] + \frac{1}{c^0}\frac{dC_{OA}}{dt}\right)}{\left(\frac{C_{OA}}{c^0} + 1\right)} c_g$$

In order to have a system of 3 differential equations, we also need to write eq. 4 as a differential equation.

$$C_{OA} = C_{SOA} + c_p \rightarrow \frac{dC_{OA}}{dt} = \frac{d(C_{SOA} + c_p)}{dt} \rightarrow$$

$$\frac{dc_p}{dt} = \frac{dC_{OA}}{dt} - \frac{dc_{SOA}}{dt}$$

S3. Organonitrates Calculation

For the organonitrates calculation we apply the formula from Kiendler-Scharr et al. (2016) which is based on the NO_2^+/NO^+ ratio. More specifically, the fraction of organonitrates to the total nitrates was calculated based on the following equation:

$$OrgNit = \frac{(1 + R_{ONit})(R_{meas} - R_{cal})}{(1 + R_{meas})(R_{ONit} - R_{cal})}$$

where R_{meas} is the measured NO_2^+/NO^+ ratio at the experiment, R_{cal} is the NO_2^+/NO^+ ratio during the calibration of the AMS with ammonium nitrate (0.88 in our case) and R_{ONit} is the minimum NO_2^+/NO^+ ratio during the calibration of the AMS (0.045 in our case).

S4. AMS results of all chemical aging experiments

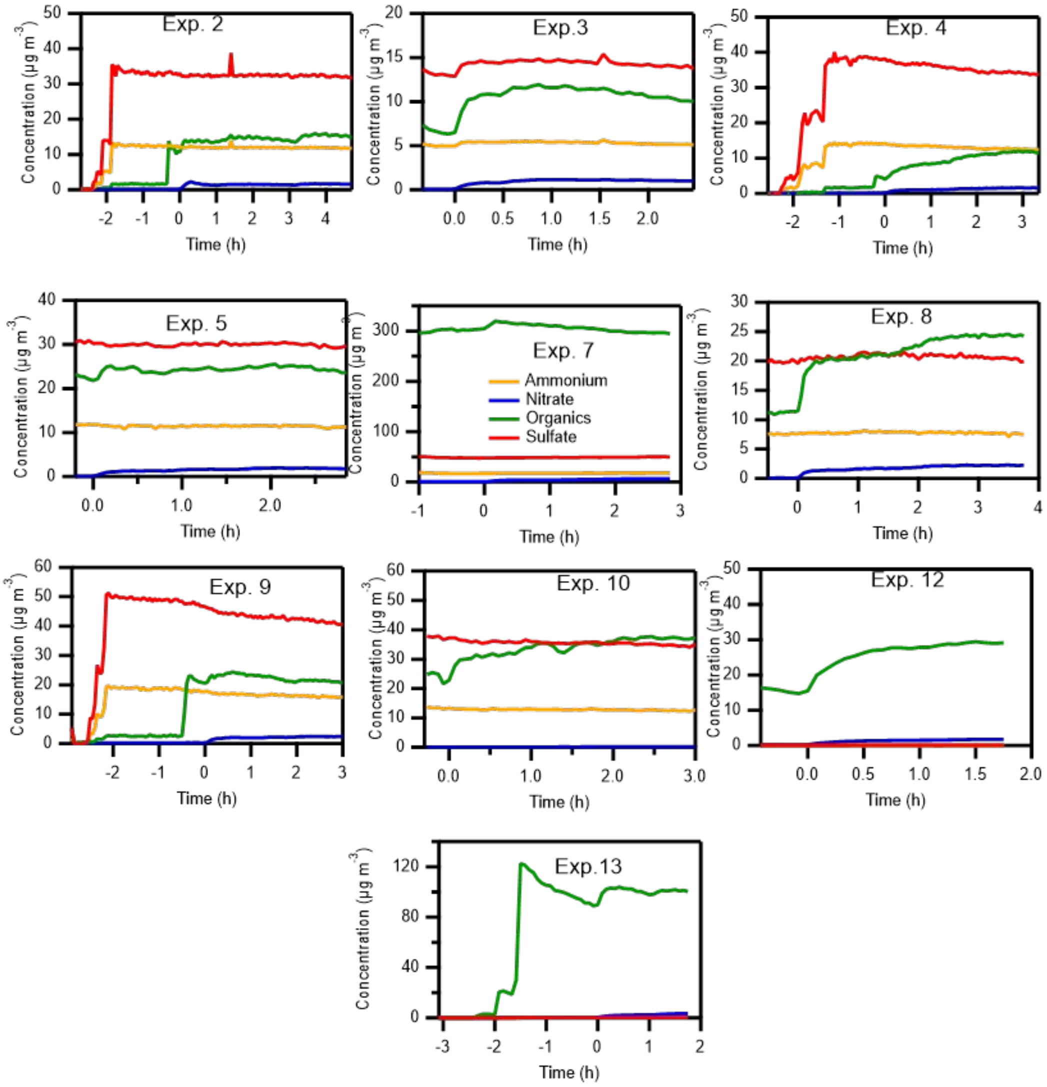

Figure S2: Time series of AMS measurements for all experiments. Time zero is the start of oxidation.

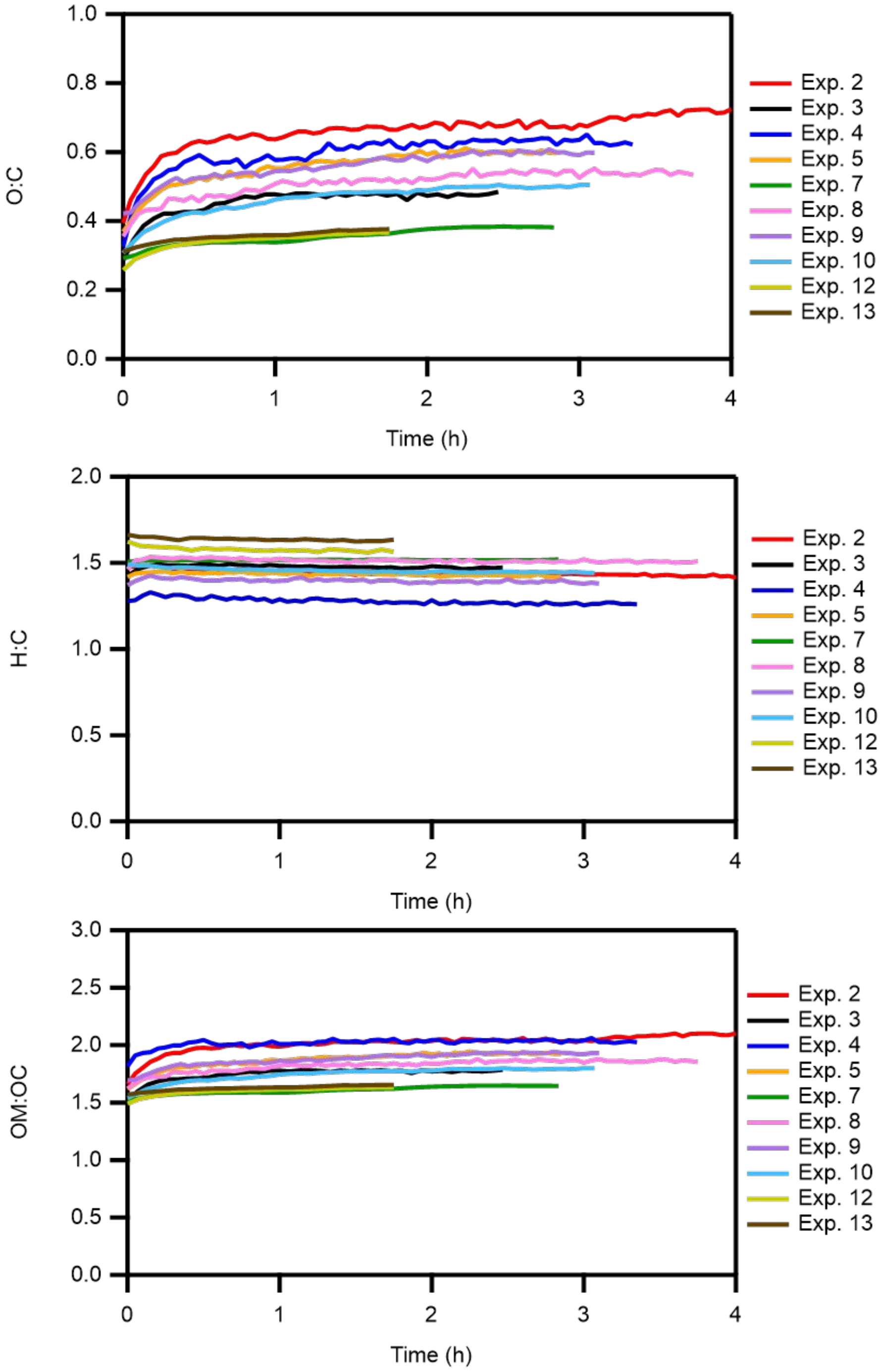

Figure S3: Time series of O:C, H:C and OM:OC of all experiments for the three compounds. Small differences are observed because of the different compounds.

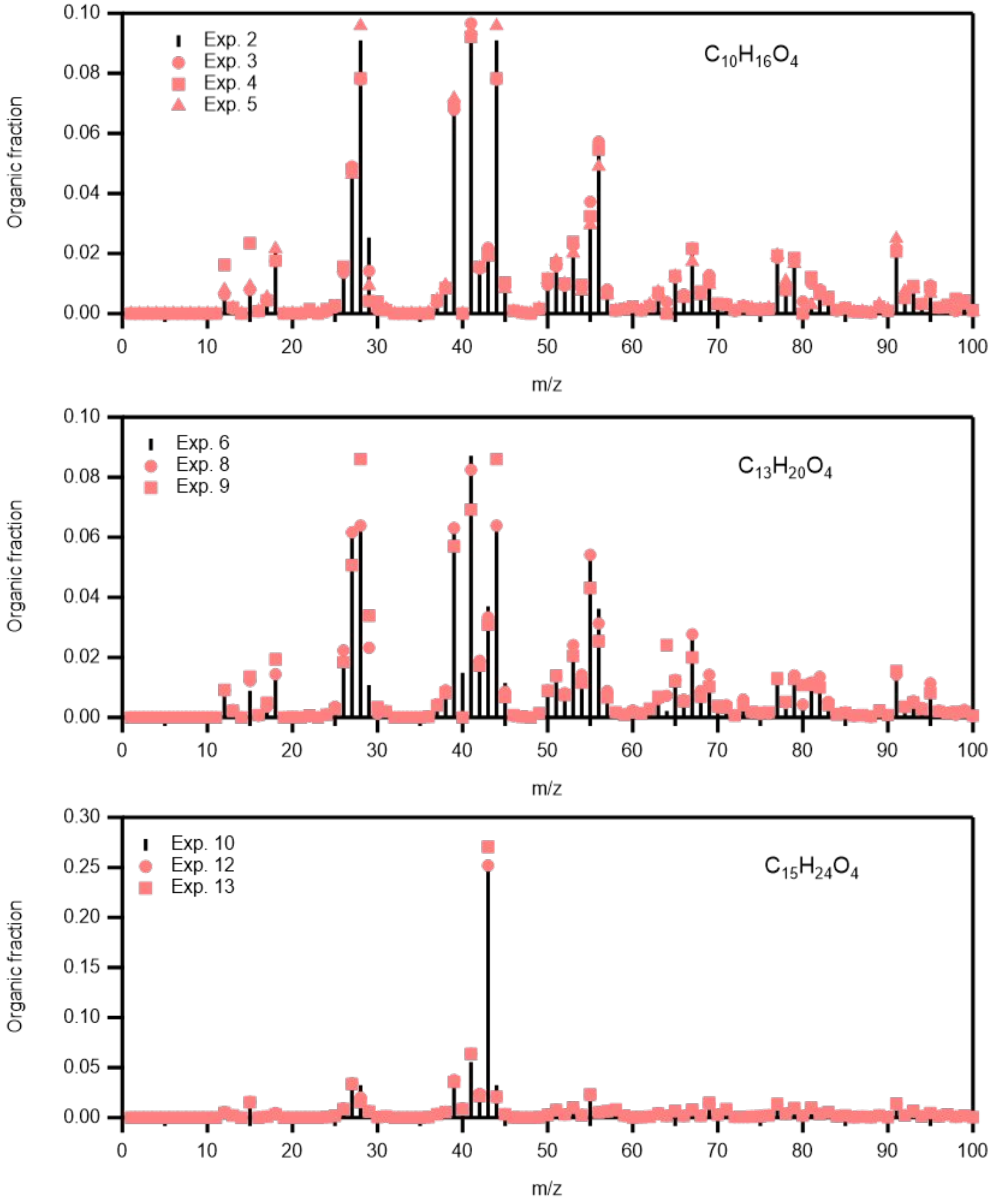

Figure S4: Fractional spectra of each compound for the different experiments.

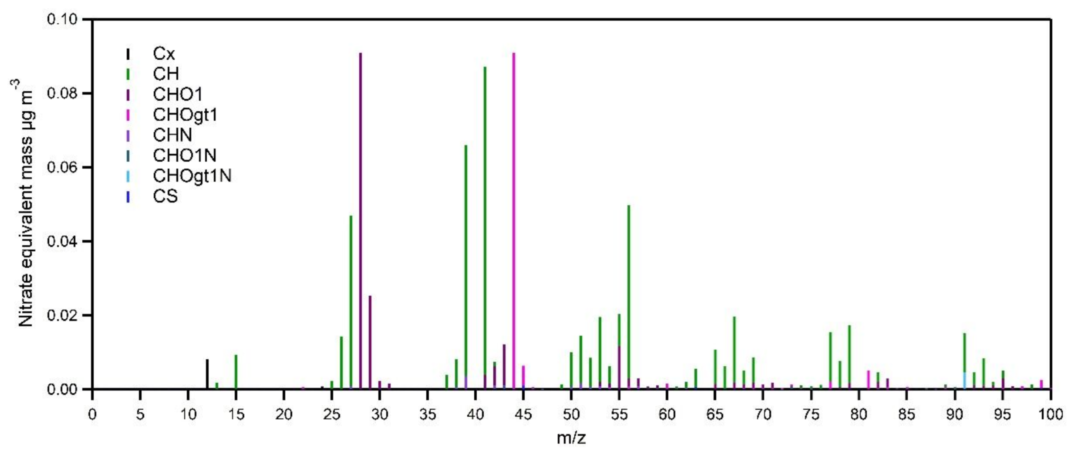

Figure S5: Organic high-resolution spectrum of the compound $C_{10}H_{16}O_4$ obtained from Exp.2.

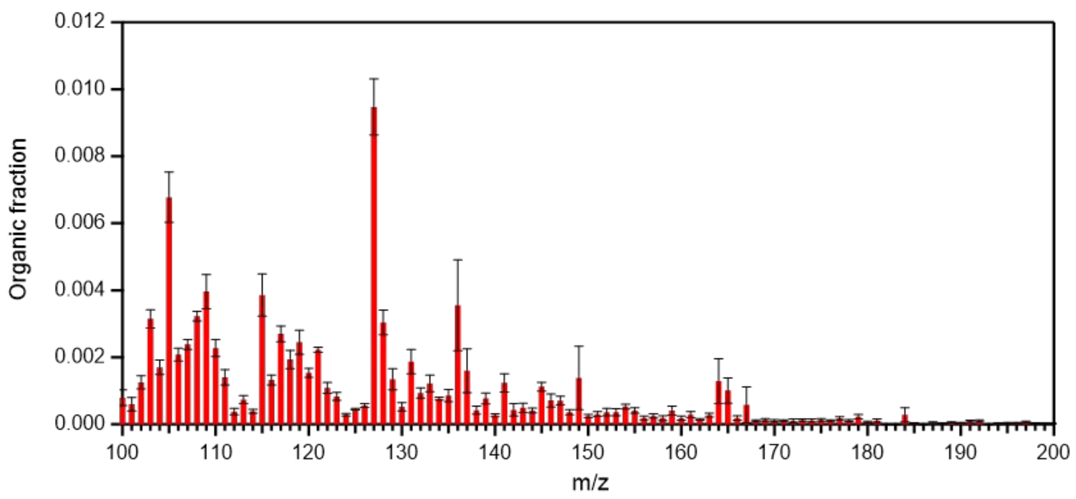

Figure S6: Average AMS spectrum of the SOA from all the $C_{10}H_{16}O_4$ oxidation experiments for the large *m/z's* (above 100). The red lines represent the average fraction of each *m/z* and the error bars represent the standard deviation from the different experiments.

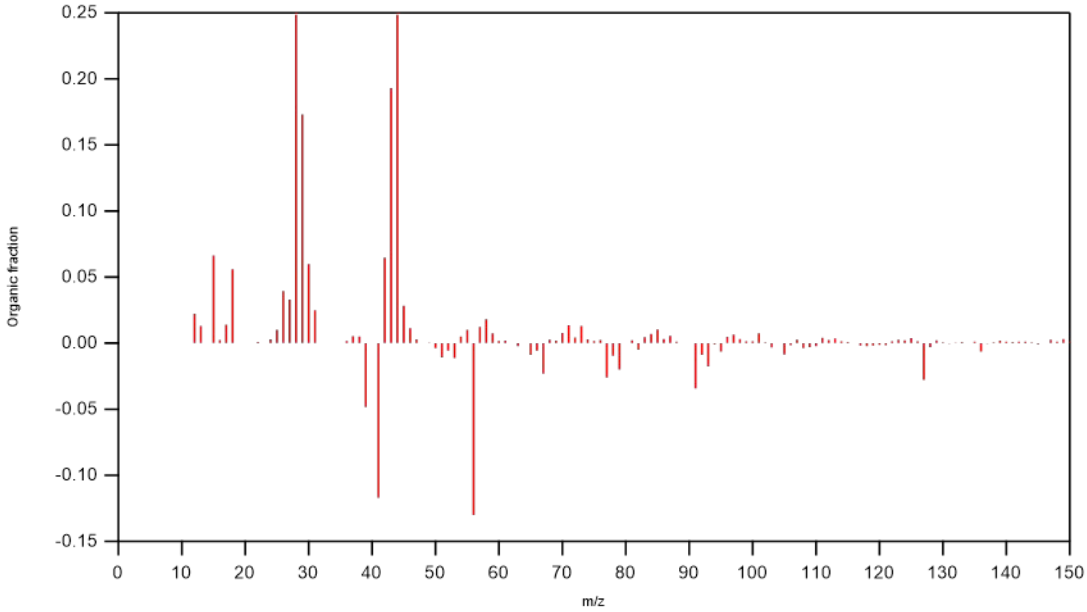

Figure S7: Difference between the initial and final OA spectra shown in Figure 5. The final spectrum was subtracted from the initial (actual mass) and then normalized to unity for visual representation.

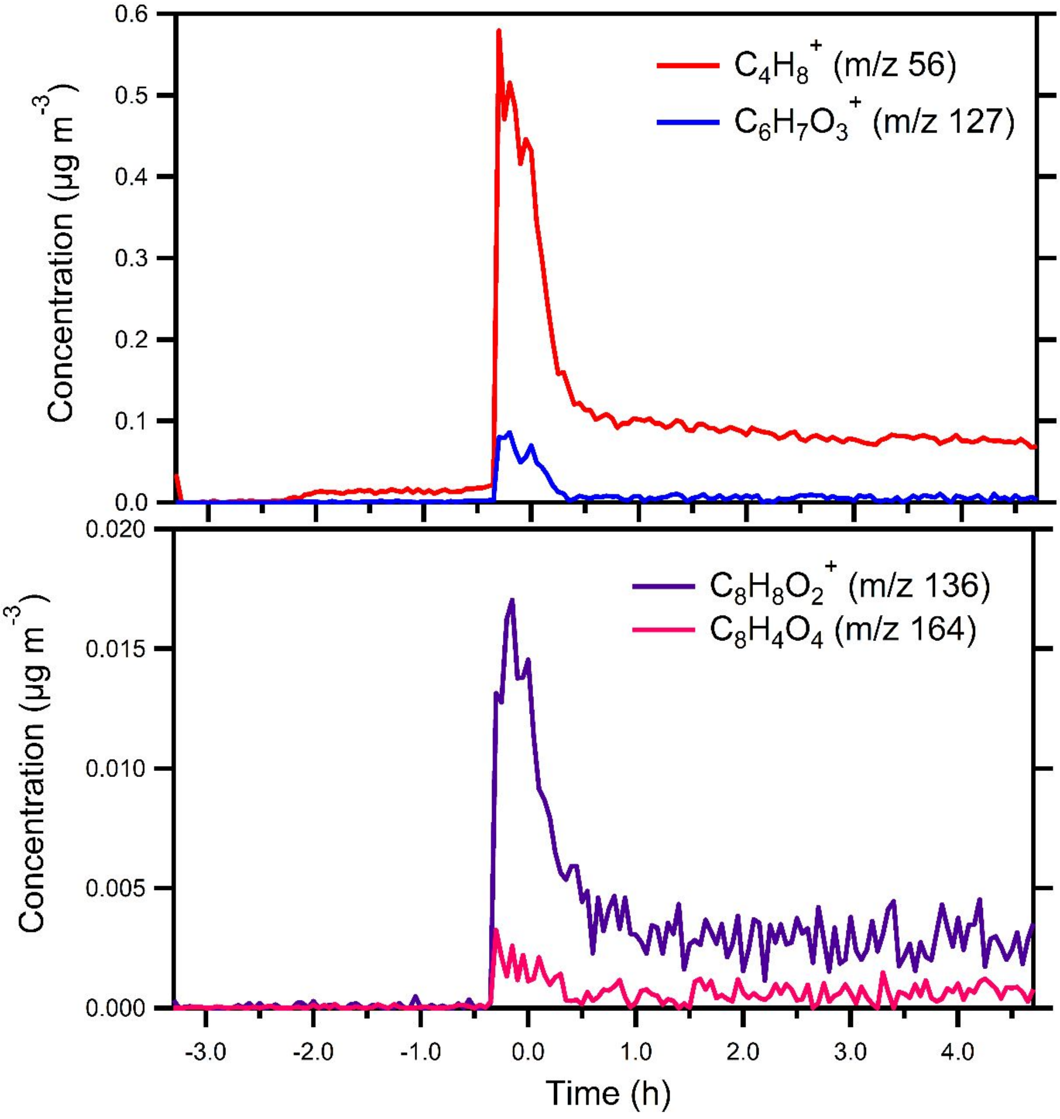

Figure S8: Timeseries of specific *m/z's* measured by the AMS which could be used as potential tracers for the $C_{10}H_{16}O_4$.

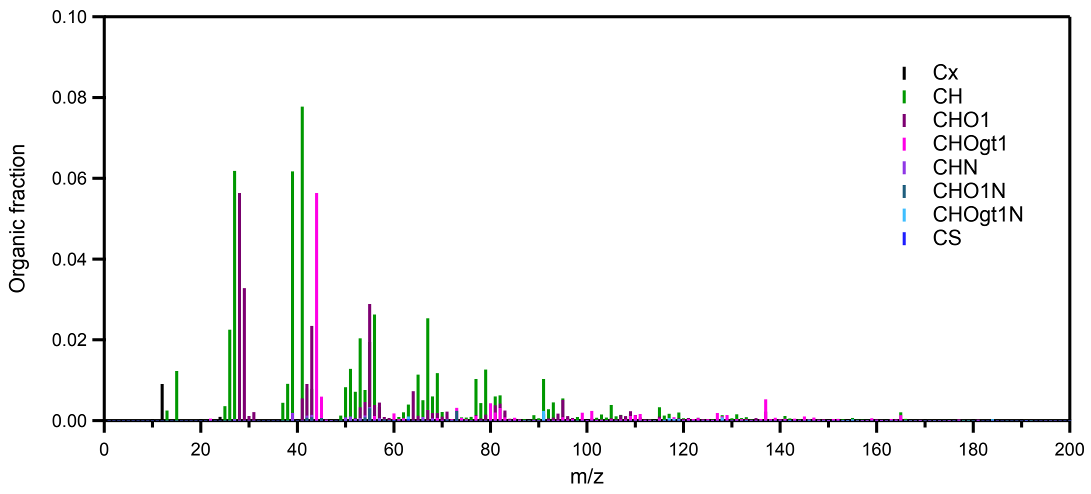

Figure S9: Organic high-resolution spectrum of the compound $C_{13}H_{20}O_5$ obtained from Exp. 8.

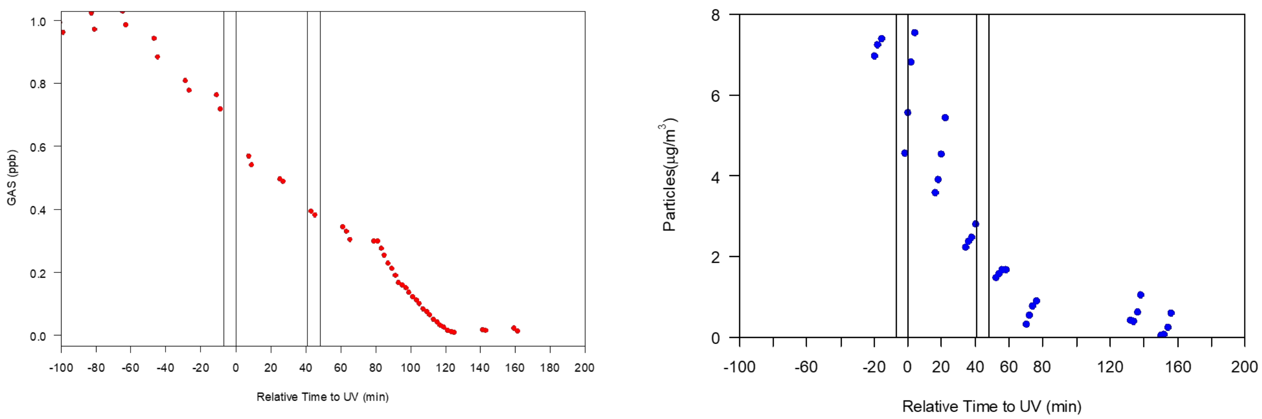

Figure S10: Gas (left figure) and particle phase (right figure) concentrations of Exp. 5 as obtained by the PTR-ToF-MS. The high-resolution PTR-MS was only available during Exp. 5. In the rest of the experiments a unit resolution PTR-MS was used.

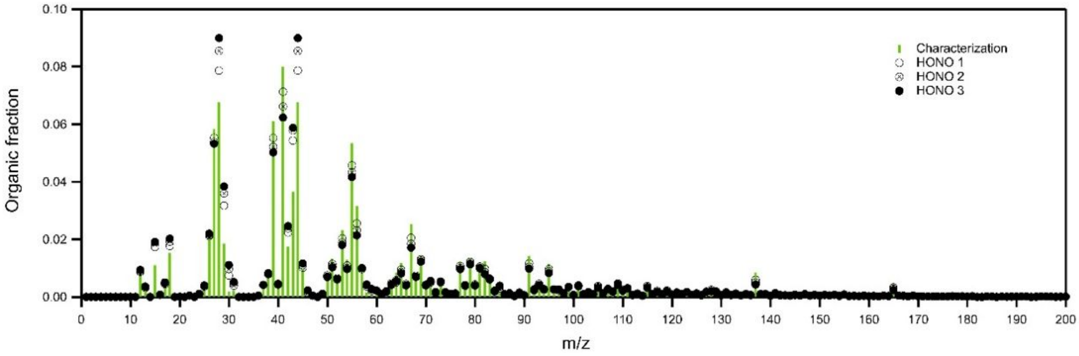

Figure S11: Comparison of the organic spectrum of the $C_{13}H_{20}O_5$ oxidation experiment at different stages of oxidation. The spectrum of the $C_{13}H_{20}O_5$ is also shown. There were 3 HONO injections in each experiment. The "HONO 1" refers to the first, the "HONO 2" to the second and the "HONO 3" to the third. In all experiments, the gas phase reaction is relatively fast (during the first hour all the parent compound has been reacted) so the additional oxidation stages resulted in only small changes compared with the first.

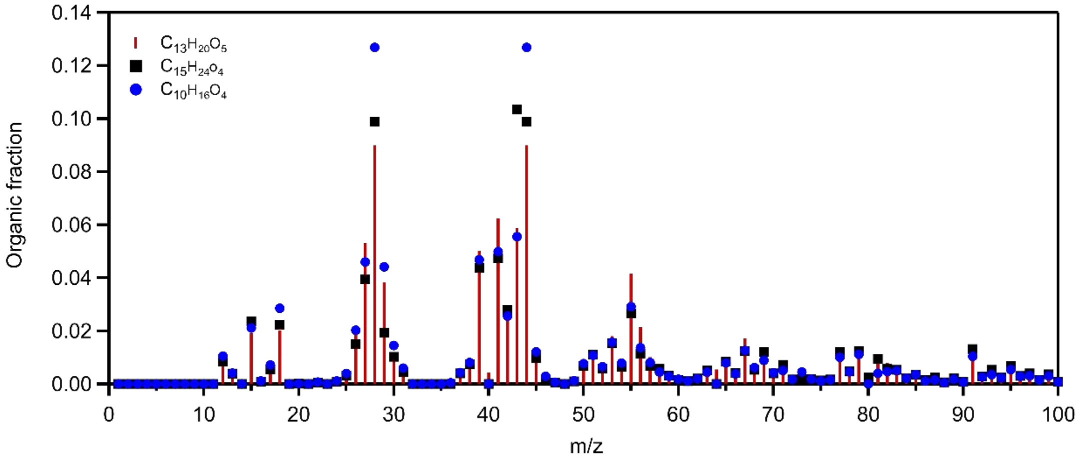

Figure S12: Comparison of the final SOA AMS spectrum from the photooxidation of the different sesquiterpenes' products. These are the average spectra of each compound at the end of all experiments

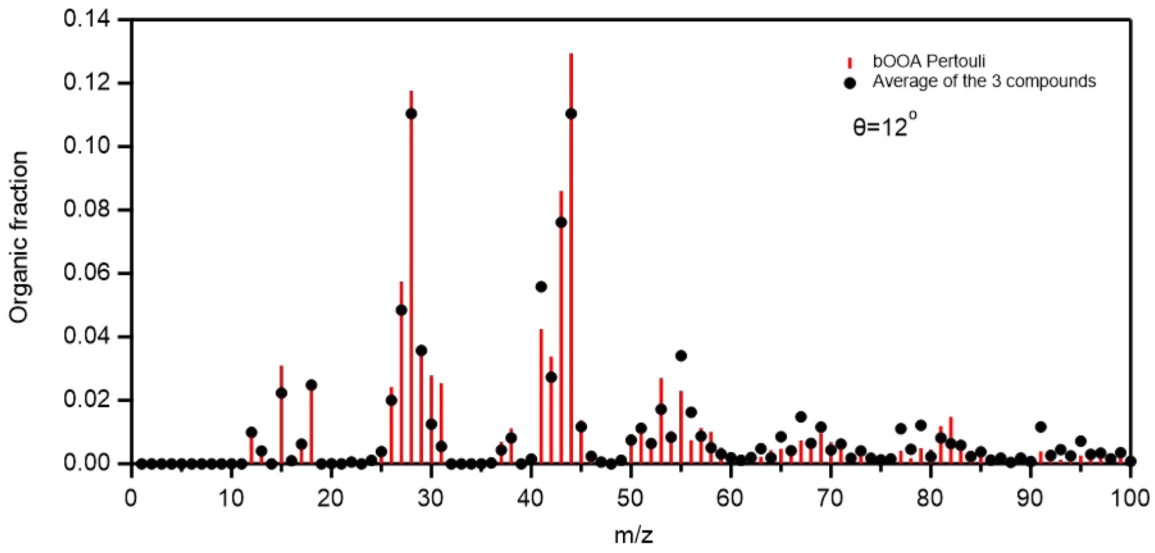

Figure S13: Comparison of the bOOA spectrum from SPRUCE-22 field campaign in Pertouli with the average SOA spectrum from the photooxidation of the sesquiterpenes' products.

Table S1: OH exposure and NO_x concentration in each experiment

	OH exposure (molec s cm^{-3})	NO_x (ppb)
Exp. 2	5.4 $x10^{11}$	185
Exp. 3	4.6 $x10^{11}$	244
Exp. 4	4 $x10^{11}$	146
Exp. 5	3.1 $x10^{11}$	189
Exp. 7	3.1 $x10^{11}$	178
Exp. 8	5.4 $x10^{11}$	142
Exp. 9	2.1 $x10^{11}$	-
Exp. 10	4.8 $x10^{11}$	102
Exp. 12	1.9 $x10^{11}$	116
Exp. 13	2.1 $x10^{11}$	198

*The NO_x concentration refers to the average concentration after 1h of oxidation. In Exp. 9 the NO_x monitor was unavailable due to technical issues.

S5. PMF analysis of chemical aging experiments

PMF analysis was performed for each chemical aging experiment separately in order to distinguish the precursor from later-generation secondary organic aerosol (SOA). No a-priori information about the factor profiles was used. These compounds have not been studied before so their spectra are not known. OA high-resolution mass spectra were analysed (*m/z* 12-300). The high-resolution results were summed to unit resolution after the analysis for presentation purposes. The error matrix of the organics was calculated

by PIKA and was used as an input to the PMF. The “weak” signals (signal-to-noise (S/N) ratio between 0.2 and 1) were downweighed by a factor of 2 and the “bad” (S/N below 0.2) were removed. Also, the variables related to CO_2 and CO_2-based corrections (16, 17, 18, 28, and 44) were downweighed by a factor of 2. The rotational ambiguity of the solution was explored using the Fpeak approach. The minimum Fpeak value was −1, the maximum was 1, and the step was 0.1. The PMF results of all experiments are shown in the figures below.

The PMF results retrieved successfully the precursor factor, the SOA factor and an additional factor which was consistent with impurities introduced into the chamber with the seeds. The 4-factor solution reduced the Q/Qexp only marginally. Also, the factor 4 spectrum was not different from the rest, showing that the 3-factor solution was numerically satisfactory. In the beginning of the experiment only impurities were present, so this factor was well constrained. After the injection of $C_{10}H_{16}O_4$ the AMS spectrum should be dominated by the signal from this factor. After the beginning of the oxidation the concentration of $C_{10}H_{16}O_4$ should decrease during the experiment, while the concentration of the later generation SOA should decrease. Taking into account the above characteristics of our experiments the assignments of the factors were relatively straightforward. There was no reason to use information about known AMS spectra (which are not available in any case for the corresponding compounds).

In the unseeded experiments (Exps. 12-13) the PMF analysis was able to explain the results with only 2 factors, so it is consistent with our explanation about the source of the impurities. Different ammonium sulfate solutions were prepared for different experiments and different trace impurities could be present in the aerosol generation system. These differences can also be partially explained by the high uncertainty of the PMF analysis at these low concentration levels, and the small differences in their concentration. The impurities were a small fraction of the total OA in each experiment. Concerning the impurities spectrum, it was dominated by the *m/z* 29 (CHO^+) (15N correction was performed). However, these are fractional contributions of low concentration data which can lead to uncertain impurity spectra. The impurities concentration was subtracted from the OA concentration in the model.

The behavior of the impurities at time zero (Fig. 7) is mainly due to mixing effects during the introduction of the precursor (and the associated clean air) at a point in the chamber during the sampling point. This incomplete mixing is also observed in the precursor concentration measurements (they decreased for a few minutes and then they increased). On the other hand, in the C13 experiment the system reached uniform mixing faster based at least on the behavior of the precursor concentration. The uncertainty of the PMF analysis could also be contributing to the estimated behavior of the corresponding PMF factor.

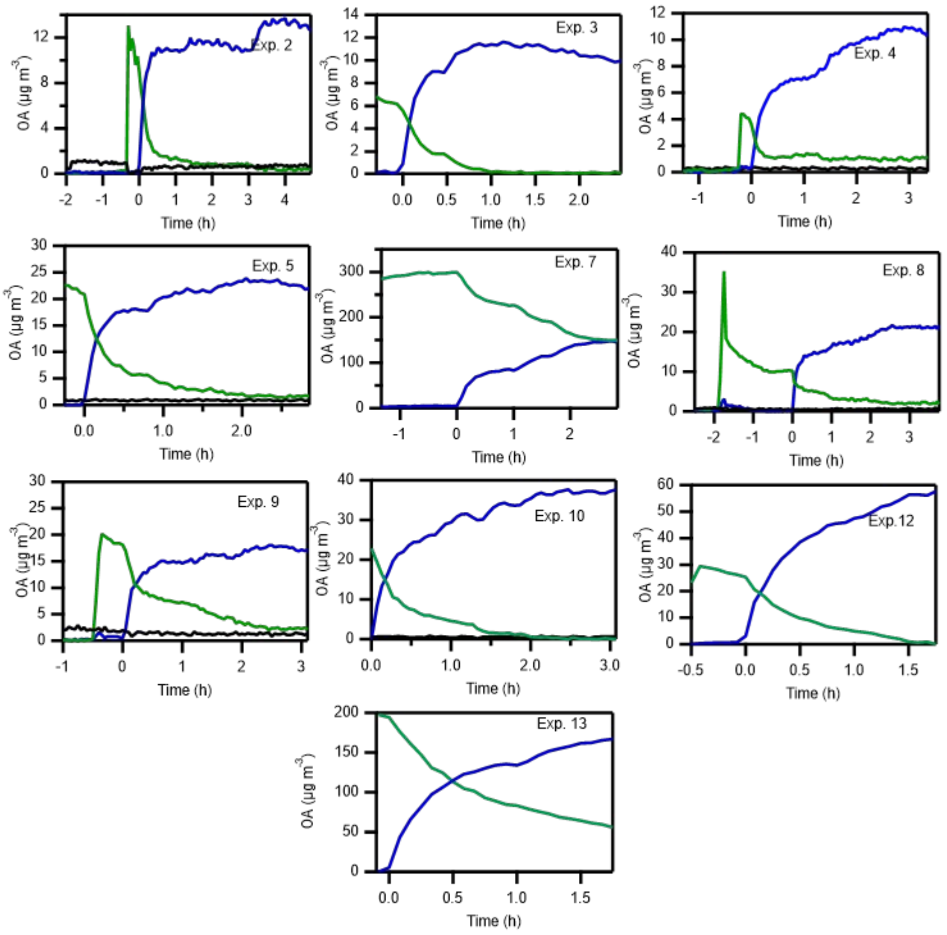

Figure S14: Time series of the PMF factors of all experiments. The green line represents the precursor, the blue line represents the later generation SOA and the black lines represent the impurities introduced inside the chamber together with the ammonium sulfate seeds. Impurities are a minor component of the OA in each case. In experiments 12 and 13 there were no seeds injection, and as a result no impurities.

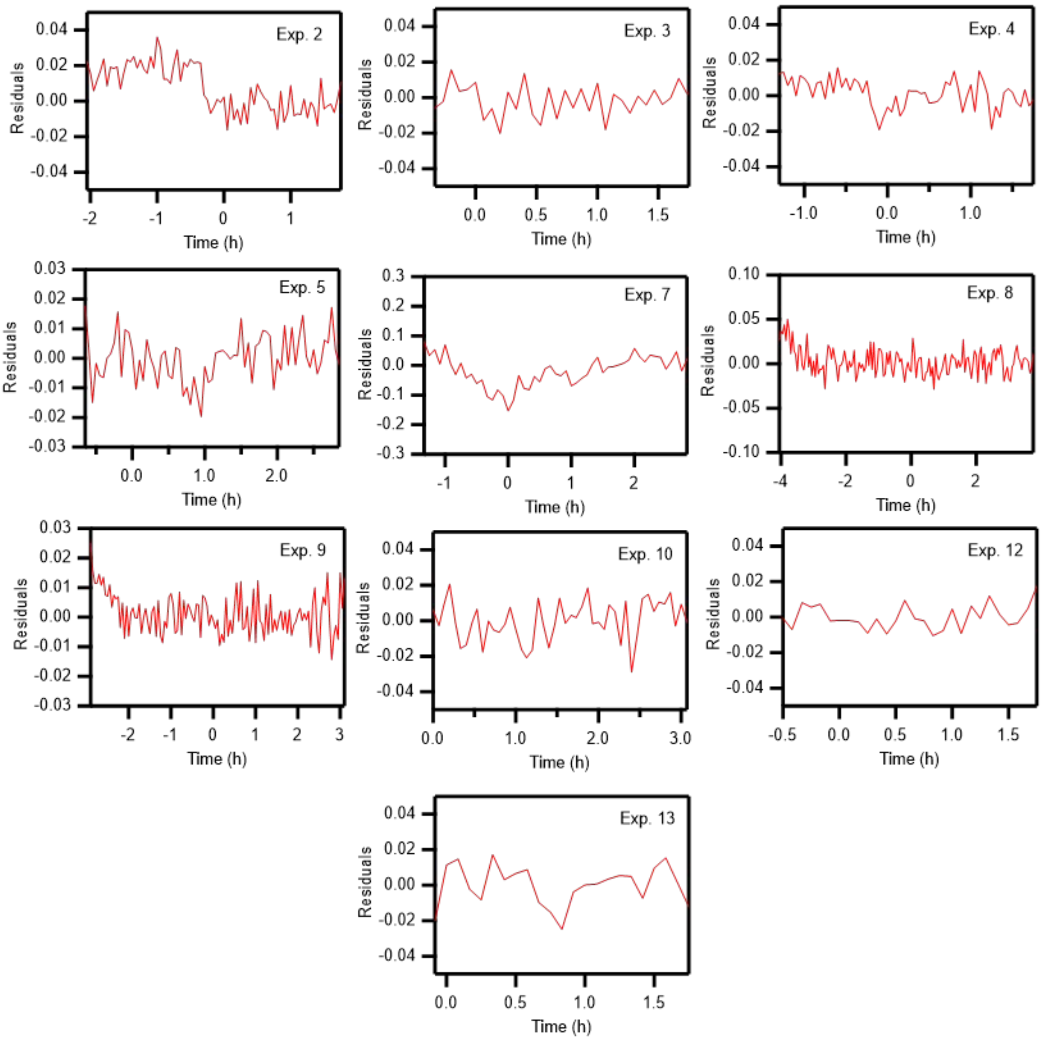

Figure S15: Residuals timeseries of the PMF solution of each experiment.

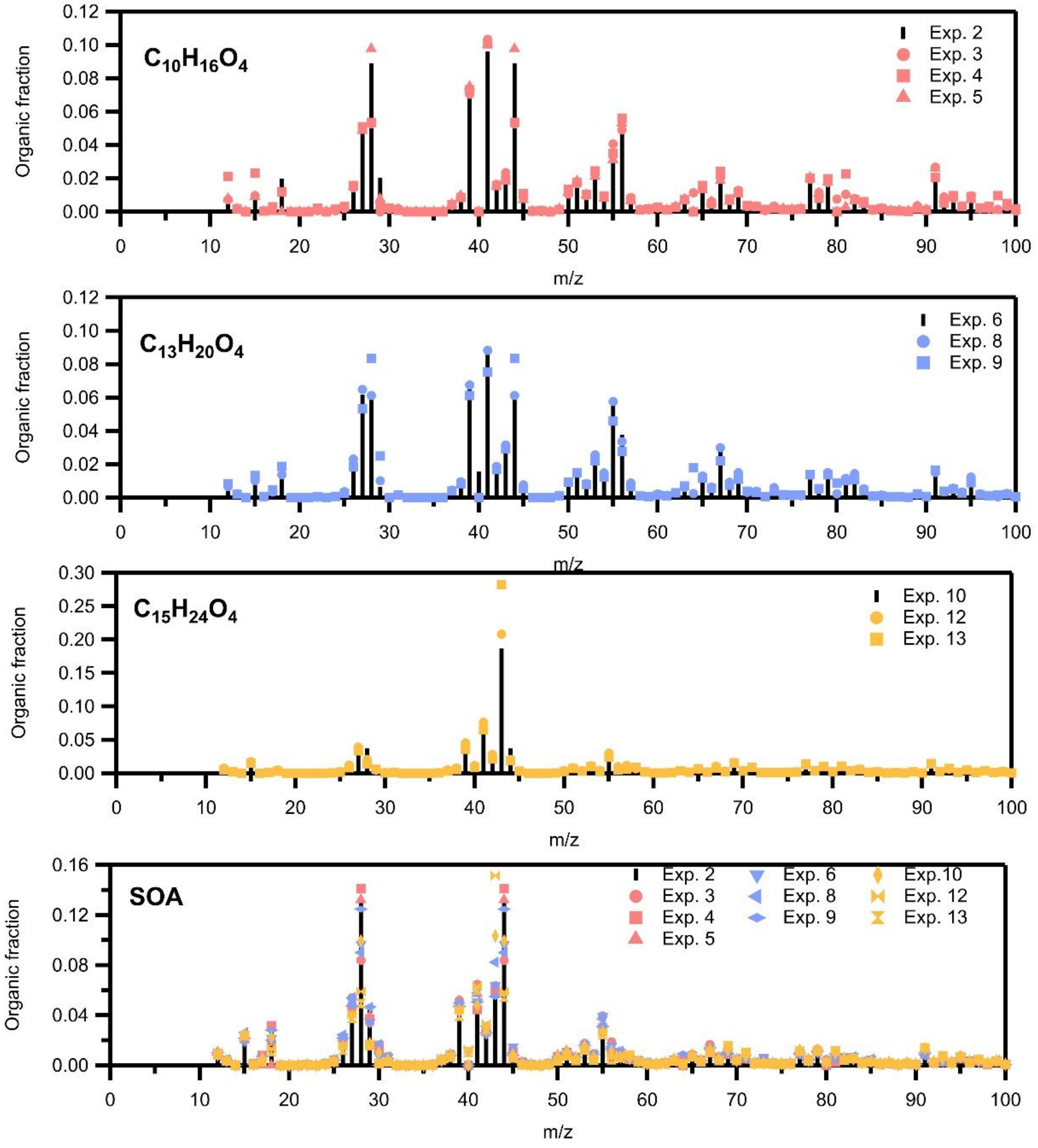

Figure S16: PMF factor spectra of all experiments.

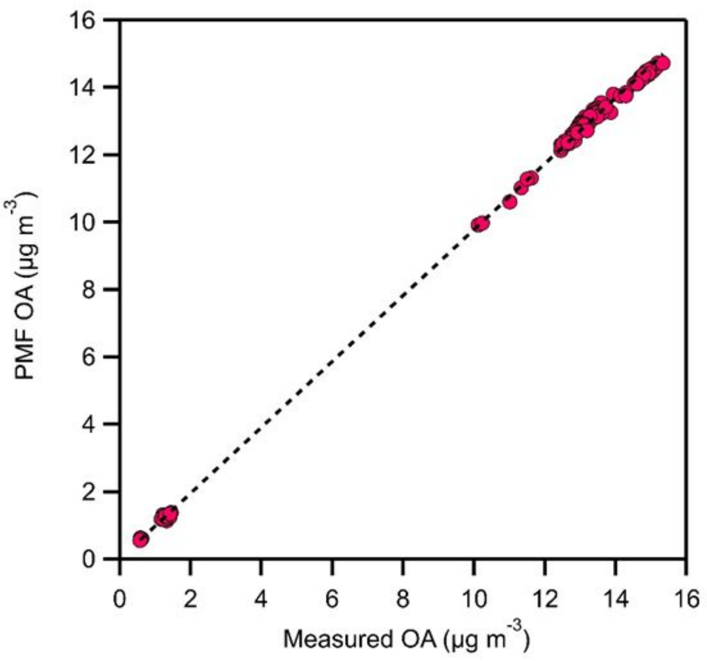

Figure S17: Comparison between the measured OA concentration (x-axis) and the PMF reconstructed OA concentration (y-axis). The dashed lines correspond to the linear fit of the data points. The R^2 is 0.99.

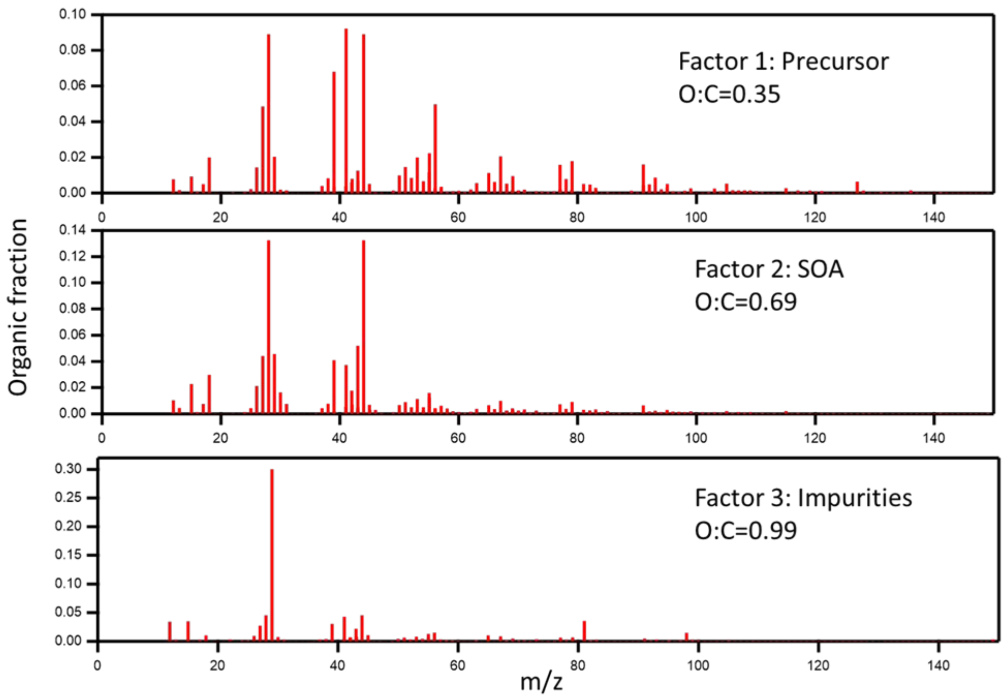

Figure S18: Organic mass spectra of the three factors obtained from the PMF analysis of the $C_{10}H_{16}O_4$ photooxidation experiment (Exp. 2).

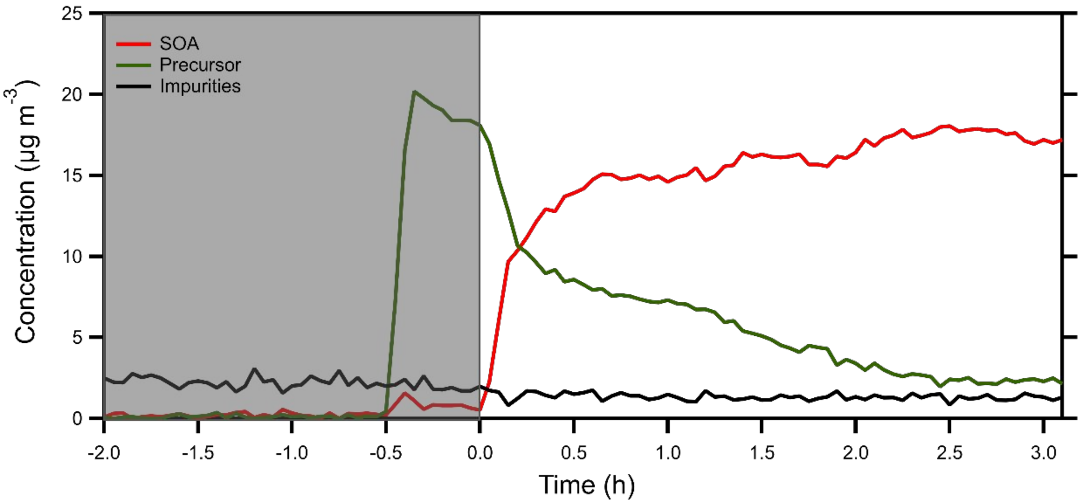

Figure S19: Time series of the factors obtained from PMF from the $C_{13}H_{20}O_5$ oxidation experiment (Exp. 9).

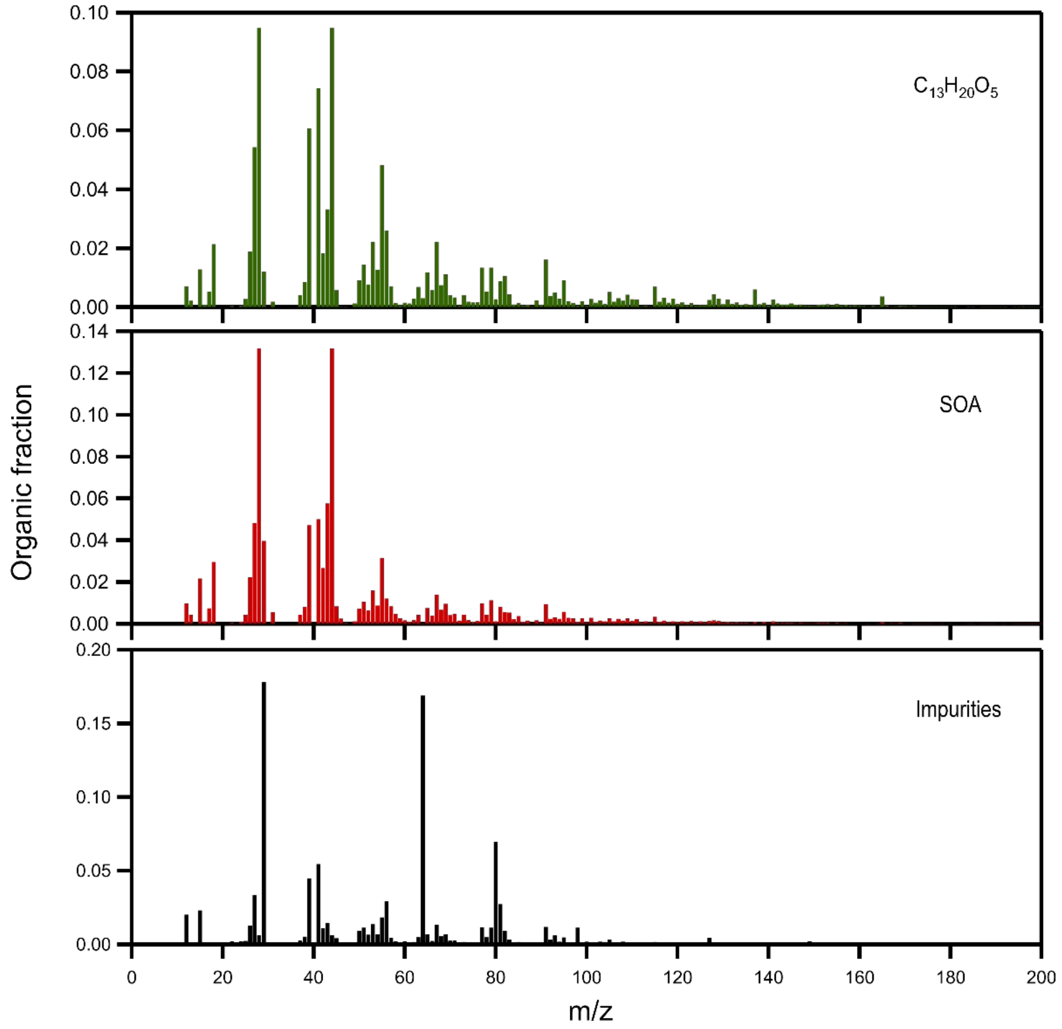

Figure S20: Organic mass spectra of the three factors obtained from the PMF analysis of the $C_{13}H_{20}O_5$ photooxidation experiment (Exp. 9).

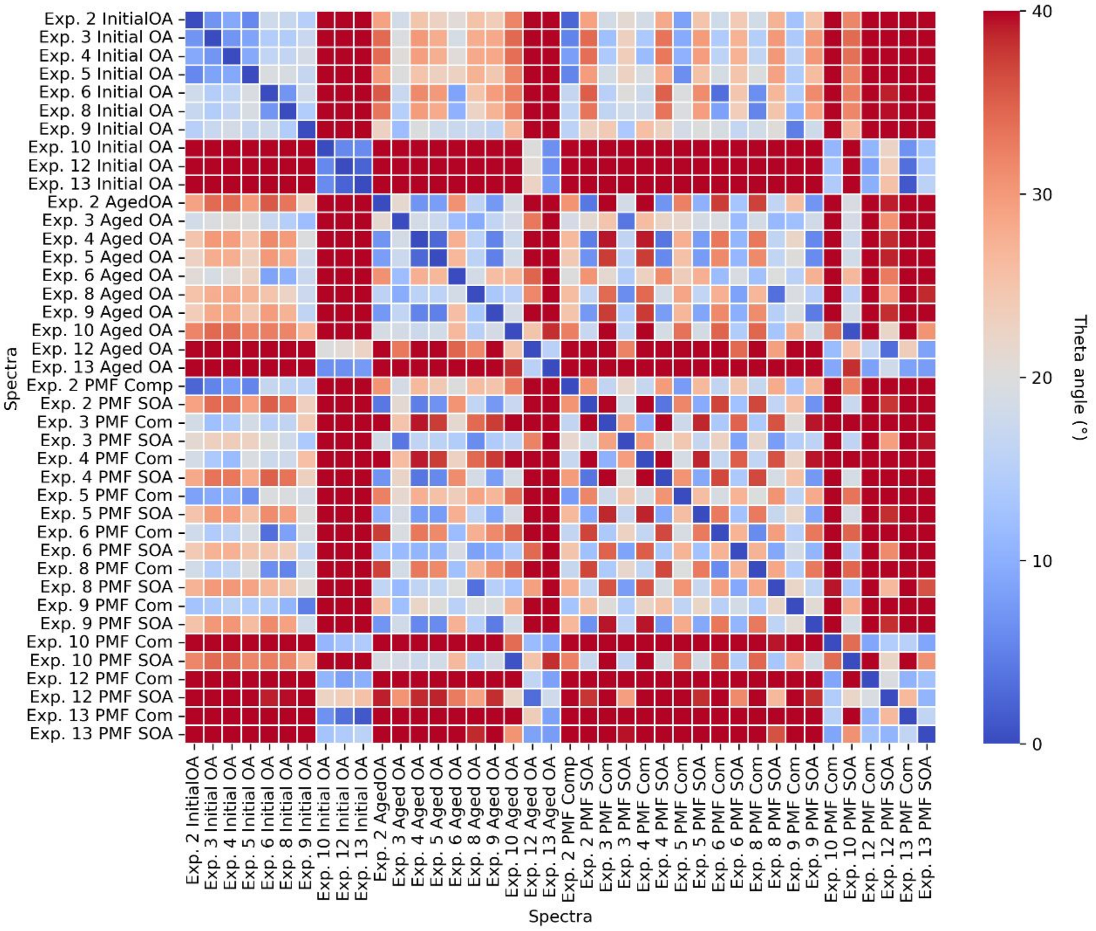

Figure S21: Theta angles among all spectra. The initial and final OA in each experiment are shown as "initial OA" and "aged OA" and the PMF factors are shown as "PMF Com" for the compound factor and "PMF SOA" for the later generation SOA factor for each experiment.

References

Kiendler-Scharr, A., Mensah, A.A., Friese, E., Topping, D., Nemitz, E., Prévôt, A.S., Äijälä, M., Allan, J., Canonaco, F., Canagaratna, M. and Carbone, S. Ubiquity of organic nitrates from nighttime chemistry in the European submicron aerosol. *Geophysical Research Letters*, *43*, 7735-7744, 2016.
